# Combined treatment with anti‐PSMA CAR NK‐92 cell and anti‐PD‐L1 monoclonal antibody enhances the antitumour efficacy against castration‐resistant prostate cancer

**DOI:** 10.1002/ctm2.901

**Published:** 2022-06-13

**Authors:** Fangming Wang, Liyuan Wu, Le Yin, Hui Shi, Yuchun Gu, Nianzeng Xing

**Affiliations:** ^1^ State Key Laboratory of Molecular Oncology National Cancer Center/National Clinical Research Center for Cancer/Cancer Hospital Chinese Academy of Medical Sciences and Peking Union Medical College Beijing China; ^2^ Department of Urology National Cancer Center/National Clinical Research Center for Cancer/Cancer Hospital Chinese Academy of Medical Sciences and Peking Union Medical College Beijing China; ^3^ R&D department Allife Medicine INC Beijing China; ^4^ Molecular Pharmacology Laboratory Institute of Molecular Medicine Peking University Beijing China; ^5^ Translation Medicine Research Group (TMRG) Aston Medical School Aston University Birmingham UK; ^6^ Department of Urology Shanxi Province Cancer Hospital/Shanxi Hospital Affiliated to Cancer Hospital Chinese Academy of Medical Sciences/Cancer Hospital Affiliated to Shanxi Medical University Taiyuan China

**Keywords:** CAR NK‐92 cell line, castration‐resistant prostate cancer, PD‐L1

## Abstract

**Background:**

The chimeric antigen receptor NK‐92 (CAR NK‐92) cell targeting the prostate‐specific membrane antigen (PSMA) has shown antitumour effects in castration‐resistant prostate cancer (CRPC). However, the expression changes of programmed death ligand 1 (PD‐L1) and its mechanisms on CAR NK‐92 and CRPC cells and the effect of the anti‐PD‐L1 monoclonal antibody (mAb) on PD‐L1 expressed on CAR NK‐92 cells remain unknown.

**Methods:**

Human dendritic cells and CD8^+^ T cells were acquired from blood samples of healthy donors and cocultured with C4‐2 cells. Changes in PD‐L1 expression were detected by flow cytometry. Differential gene expressions were investigated by RNA sequence analysis, while the regulation of PD‐L1 molecular signaling was explored using western blotting. In vitro cytotoxicity was evaluated using the Cell Counting Kit‐8 assay and the bioluminescent intensity (BLI) of green fluorescent protein‐labelled C4‐2 cells. CRPC growth in vivo was monitored using callipers and BLI in male NOD/SCID mice subcutaneously injected with C4‐2 cells and treated intravenously with anti‐PD‐L1/PD‐1 mAb, CAR NK‐92 or cocultured CD8^+^ T cells.

**Results:**

Significantly upregulated expression of PD‐L1k was observed in cocultured C4‐2 and CAR NK‐92 cells. In addition, upregulation of PD‐L1 expression was dependent on interferon‐γ in C4‐2 cells, while it was dependent on direct cell‐to‐cell interaction via the NK group 2 member D/ phosphatidylinositol 3‐kinase/AKT pathway in CAR NK‐92 cells. The anti‐PD‐L1 mAb directly acted on PD‐L1 expressed on CAR NK‐92 cells and augmented the cytotoxicity of CAR NK‐92 cells against C4‐2 and CRPC cells from one patient in vitro. Anti‐PD‐L1 mAb significantly enhanced the antitumour effect of CAR NK‐92 cells against CRPC cells in vivo when compared to treatment with CAR NK‐92 cells or combined with anti‐PD‐1 mAb in the absence or presence of cocultured CD8^+^ T cells.

**Conclusion:**

Combined treatment with CAR NK‐92 and anti‐PD‐L1 mAb improved the antitumour efficacy against CRPC, which is of extraordinary translational value in the clinical treatment of CRPC.

## Graphical abstract

- CAR NK‐92 is activated through specific recognition of PSMA on prostate cancer cells.
- Expression of PD‐L1 on activated CAR NK‐92 is upregulated via CAR‐triggered PI3K/AKT/mTOR pathway.
- Expression of PD‐L1 on cancer cells is upregulated via IFN‐γ‐mediated JAK‐STAT pathway.
- Atezolizumab potentiates CAR NK‐92 cytotoxicity by directly acting on PD‐L1 on CAR NK‐92 and unleashes CD8^+^ T cell via blocking PD‐L1/PD‐1 axis.

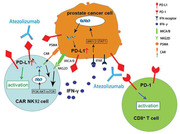

## 1 INTRODUCTION

Prostate cancer (PCa) remains the second most frequent malignancy and the fifth major cause of death from malignancy in males in 2020 worldwide.^1^ Localised PCa can be cured with radiation or surgery; however, once PCa becomes non‐organ confined, androgen deprivation therapy (ADT) is the cornerstone of therapy. But most patients will ultimately become resistant to ADT and progress to castration‐resistant prostate cancer (CRPC) after a transient response, which remains incurable.^2^, ^3^ Several studies have been conducted to evaluate the role of immunotherapeutic agents, including new immune checkpoint inhibitors (ICIs) and sipuleucel‐T.^4^ However, ICI monotherapy demonstrated limited efficacy in PCa patients, probably because of an immunologically cold tumour microenvironment (TME).^5^ Thus, T or NK cell engineered with chimeric antigen receptors (CARs) is believed to provide an alternative for the treatment of CRPC. CAR consists of an extracellular domain that harbours an antibody that can bind to tumour‐specific antigens, a transmembrane region and an intracellular signalling domain.^6^

Although CAR T cells targeting CD19 have achieved success in the treatment of lymphoid malignancies in clinical settings,^7^ the use of CAR T cells in treating solid tumours is limited owing to the complicated TME and severe immune‐related adverse events. CAR NK cells have obvious advantages over CAR T cells, such as better safety, the multiplicity of their cytotoxic mechanisms and high feasibility for ‘off‐the‐shelf’ manufacturing.^8^ We successfully constructed CAR NK‐92 cells that can specifically recognize prostate‐specific membrane antigen (PSMA) on PCa, especially CRPC cells, and proved that these effector cells possess powerful specific antitumour functions in vivo. However, CAR NK‐92 cells secrete abundant interferon‐γ (IFN‐γ) when they kill tumours, and IFN‐γ has been reported to not only generate a polarised immune response but also promote adaptive immune resistance by upregulation of programmed death ligand 1 (PD‐L1) on tumour cells in the TME.^9^ The PD‐L1 upregulation of CRPC cells could be considered a side effect of CAR NK‐92 cell treatment because PD‐L1 binding to T cells leads to the exhaustion of T cells. Therefore, we hypothesised that an anti‐PD‐L1 monoclonal antibody (mAb) or anti‐programmed death 1 (anti‐PD‐1) mAb would reverse this immune suppression, reinvigorate the CD8^+^ T‐cell activity and enhance the antitumour effect on CRPC when combined with CAR NK‐92 cell therapy.

Moreover, the expression of PD‐L1 was found in immune cells besides tumour cells, and its function in immune cells has attracted considerable attention in recent years.^10^, ^11^, ^12^, ^13^, ^14^ For example, T‐cell expression of PD‐L1 has tolerogenic effects on tumour immunity by restraining macrophages and effector T cells.^10^ Furthermore, PD‐L1 delivers a constitutively negative signal to macrophages; PD‐L1 antibodies increase their proliferation, activation and survival and trigger macrophage‐mediated antitumour activity.^11^ PD‐L1 blockade can reinvigorate dendritic cells (DCs) to prime T cells in patients with cancer.^12^ NK expression of PD‐L1 can limit DC activation by binding to PD‐1 on DCs, thus reducing CD8^+^ T‐cell priming in the TME.^13^ PD‐L1 was reported to be a marker of NK‐cell activation, and NK cells expressing PD‐L1 showed enhanced antitumour activity, which could be further activated by atezolizumab against PD‐L1‐ tumours independent of PD‐1.^14^ However, the exact mechanisms underlying the regulation of PD‐L1 expression in CAR NK‐92 cells remain unknown. It also remains unexplored whether anti‐PD‐L1 mAb therapy could influence the antitumour function of CAR NK‐92 cells in the TME.

In the current study, we explored the changes in PD‐L1 expression and its mechanisms in cocultured CAR NK‐92 and CRPC cells, including a PCa cell line and primary cells from one patient with CRPC, and the effect of an anti‐PD‐L1 mAb on the antitumour activity of CAR NK‐92 cells in the absence or presence of CD8^+^ T cells. Our research provides valuable clues about the molecular mechanisms of PD‐L1 regulation as well as its role in immune cells, which could be used for immunotherapeutic strategies for CRPC.

## 2 METHODS

### 2.1 Cell lines

NK‐92, C4‐2 (a human CRPC cell line expressing PSMA), and 293T cell lines were purchased from the American Type Culture Collection. C4‐2 cells were cultured in RPMI‐1640 medium (Servicebio) supplemented with 10% fetal bovine serum (FBS) (Biological Industries). Unirradiated or irradiated NK‐92 and CAR NK‐92 cells were incubated in an alpha minimum essential medium (alpha MEM; Gibco) supplemented with 20% FBS, 200 U/ml recombinant human IL‐2 (SL Pharm), 0.1 mM β‐mercaptoethanol (PAN‐Biotech), 0.02 mM folic acid (Sigma) and 0.2 mM inositol (Sigma). 293T cells were cultured in Dulbecco's modified Eagle medium (DMEM; HyClone) or Opti‐MEM (Gibco).

### 2.2 Construction of CAR‐expressing NK‐92 cells

We developed a CAR construction targeting human PSMA based on a novel selected high‐affinity specific polypeptide. The amino acid sequences of the three peptides used for CAR construction are listed as follows: KHLHYHSSVRYG (KHL), WTNHHQHSKVRE (WTN) and GTIQPYPFSWGY (GTI). Each peptide segment was crossed with two repeats, and the interpeptide segments were separated using a linker. The intracellular signalling domains of CAR were generated by fusing full‐length human NK group 2 member D (NKG2D) to the 2B4 (CD244) signalling domain of the natural killer cells. We then used the CAR lentiviral transfer vector to produce viral particles in 293T cells as previously described.^15^, ^16^ After the transduction of CAR by viral particles, NK‐92 cells modified with CAR were selected and cloned. We proved that the CAR NK‐92 cells after irradiation have a limited lifespan and possess strong and specific cytotoxicity against PSMA‐expressing PCa cells (Supplementary Material, including Figures S1–S5). The CAR NK‐92 cells used in our research were irradiated with 5 Gy using an X‐ray irradiator (Varian Unique) before use.

### 2.3 Flow cytometry

After coculture, CAR NK‐92 cells were directly collected from the medium, and fluorescence‐activated cell sorting (FACS; WOLF Cell Sorter; Nano Cellect) was used to collect viable cells with negative staining of propidium iodide (PI; Thermo Fisher Scientific). C4‐2 cells were harvested from the bottom of the plates. To validate that C4‐2 cell upregulation of PD‐L1 is dependent on IFN‐γ, we stimulated C4‐2 cells with IFN‐γ concentration gradients of 0.2, 1, 5, 15 and 20 ng/ml. In addition, 40 μg/ml IFN‐γ mAb (Invitrogen, XMG1.2) was used before coculture to neutralize IFN‐γ. The harvested cells at 1 × 106/100 μl were immunostained for 20 min in the dark at 25°C with the following optional antibodies purchased from BioLegend: PD‐L1 APC, PD‐1 FITC, NKG2D FITC, MICA/B Alexa Fluor 488 and CD107a APC. Cells were also stained with the corresponding isotype antibodies as controls to ensure the specificity of immunostaining. The cells were analysed using a flow cytometer (Merck, easyCyte HT) and FlowJo software (version 10; Treestar).

### 2.4 Cytokine release assay

Briefly, 3 × 10^6^ C4‐2 cells were plated in a 10‐cm culture plate (Corning). After 12 h, 3 × 10^6^ CAR NK‐92 cells with or without atezolizumab or nivolumab were added in replaced fresh NK‐92 medium. At 2, 6, 12 and 24 h, the medium was centrifuged, and the supernatant was collected. The concentrations of IFN‐γ in the supernatant were measured using a human IFN‐γ ELISA Kit (Invitrogen). CAR NK‐92 cells without coculture were used for comparison. The curve fitting analysis of the optical density (OD) results was performed using a four‐parameter logistic nonlinear regression model.

### 2.5 Trans‐well assay

A trans‐well device (6‐well plate Tissue Culture Plate Insert, LABSELECT, 24‐mm diameter, 8‐μm polycarbonate membrane) was used to test PD‐L1 expression on CAR NK‐92 cells incubated with or without direct contact with C4‐2 cells. Then, 5 × 10^5^ C4‐2 cells were plated with 2 ml of medium in a CO_2_ incubator for 12 h. Next, 5 × 10^5^ CAR NK‐92 cells were added separately to the trans‐well chamber (blocked by the membrane from direct contact with C4‐2 cells) and the external space (direct contact with C4‐2 cells) with fresh medium. At 6 and 12 h after coculture, CAR NK‐92 cells from both regions were harvested and sent for flow cytometry measurements. Uncocultured CAR NK‐92 cells were used for comparison.

### 2.6 RNA sequencing and analysis

Viable cells were collected using FACS with negative PI staining. At least 3 × 10^6^ cells were collected from each cell type. According to previously reported methods,^17^, ^18^, ^19^, ^20^ total RNA was extracted, and cDNA libraries were constructed using KC‐Digit RNA technology (Seq Health Tech Co., Ltd.). Sequencing was performed on an Illumina NovaSeq 6000, and the statistical quantification was conducted using the feature counts software (Subread‐1.5.1; Bioconductor). A false discovery rate corrected *p* < .05 and a fold‐change > 2 were used to judge the statistically significant differences in gene expression. Kyoto Encyclopedia of Genes and Genomes (KEGG) enrichment analysis for signalling pathways was performed using KOBAS software (version 2.1.1). A corrected *p* < .05 was considered statistically significant.

### 2.7 Western blotting analysis

We incubated effector cells with C4‐2 cells in the presence or absence of an NKG2D blocker (clone 1D11, BioLegend) at 50 μg/ml, the phosphatidylinositol 3‐kinase (PI3K) inhibitor wortmannin (S2758; Selleckchem) at 10 μmol/L, and the AKT inhibitor afuresertib (S7521; Selleckchem) at 10 μmol/L and then measured the expression of downstream molecules and PD‐L1 in effector cells. Viable cells were collected as previously described, and protein was extracted using radioimmunoprecipitation assay lysis buffer, the concentrations of which were routinely measured. Cell lysates were loaded, separated and transferred to nitrocellulose membranes, which were then incubated with antibodies against PD‐L1 (1:500, Cell Signaling Technology (CST) ), PI3K (1:4000, CST), phosphorylated PI3K (p‐PI3K; 1:2000, Abcam), AKT (1:4000, CST), phosphorylated AKT (p‐AKT; 1:500, CST), mammalian target of rapamycin (mTOR; 1:1000, CST), phosphorylated mTOR (p‐mTOR; 1:1000, CST), Janus kinase 1 (JAK1; 1:1000, CST), phosphorylated JAK1 (p‐JAK1; 1:500, CST), JAK2 (1:1000, CST), phosphorylated JAK2 (p‐JAK2; 1:500, CST), signal transducer and activator of transcription 1 (STAT1; 1:1000, CST), phosphorylated STAT1 (p‐STAT1; 1:1000, CST) and β‐actin (1:5000, Immunoway). Next, the membranes were incubated with an Horse radish peroxidase (HRP)‐conjugated secondary antibody. The bands were illuminated using ECL (Millipore), and the exposed X‐ray films were screened. The integrated OD was calculated using Total Lab Quant (Version 11.5).

### 2.8 Generation of pure green fluorescent protein (GFP)‐expressing C4‐2 cells

A mixture of 15 μg GFP plasmid DNA, 5‐μg VSVG, 7‐μg PMDZG and 4‐μg Rev (Addgene) was added to 1‐ml Opti‐MEM. Next, 1 ml of Opti‐MEM and 80 μl of polyethylenimine were added. After 20 min, the 2 ml mixture was added to 293T cells at 90% confluence after DMEM (the old medium of 293T cells) was replaced with 6 ml of Opti‐MEM. After incubation in an incubator for 5 h, the Opti‐MEM was replaced with DMEM. To acquire viral particles, the supernatant was collected at 48 and 72 h, centrifuged at 5 × 10^4^ × *g* for 2 h and passed through a filter membrane (0.45 μm, Life Sciences). C4‐2 cells were seeded in 6‐well plates at 3 × 10^5^ cells/well. When confluence reached 80%, the aforementioned viral particles and 4 μg/ml polybrene were added to 2 ml of C4‐2 cell culture. After 48 h of transfection, GFP expression was visualised using a fluorescent microscope (Nikon TI‐DH), and the wells with the best transfection rates were marked. A C4‐2 cell line stably expressing GFP (C4‐2^GFP^) was established using single‐cell cloning. First, puromycin was added to each plate at 2 μg/ml to eliminate non‐GFP‐expressing C4‐2 cells. Second, the selected C4‐2 cell was adjusted to 2 × 10^4^ cells/ml, 6 μl of which was added to 10‐ml medium, and 100 μl was used to isolate one cell from the mixture (6 × 10^–3^ × 2 × 10^4^ cells/10 ml = 1.2 cells/100 μl). The 96‐well plate was observed under a fluorescent microscope, and the wells with only fluorescent cells were marked. Upon reaching 90%–100% confluence, the pure GFP‐expressing C4‐2 cells were transferred to larger cell culture flasks.

### 2.9 Bioluminescent intensity (BLI) in C4‐2^GFP^ cells

C4‐2^GFP^ cells were plated at 5 × 10^4^ cells/ml in a glass‐bottomed 2‐cm confocal plate (Biosharp Life Sciences) containing 2 ml of medium. C4‐2^GFP^ and effector cells were cocultured with atezolizumab at 10, 20 or 40 μg/ml or nivolumab at 20 μg/ml. After 12 h, the bottom of the dish was washed gently with PBS, and then the BLI was measured using an IVIS spectrum CT (Calliper Life Sciences). The excitation and emission spectra were set at 465 and 520 nm, respectively. Living Image R4.3.1 software was applied for data analysis.

### 2.10 CCK‐8) assay

A CCK‐8 (Dojindo) was used to evaluate in vitro cytotoxicity. PCa cells were seeded in 96‐well plates at 10 000 cells/well with 200‐μl culture medium and cocultured with effector cells at two different effector‐to‐target ratios (E/T) (E/T = 1:1 and 5:1) with atezolizumab (20 μg/ml) or nivolumab (20 μg/ml). The plates were supplemented with 20 μl of CCK‐8 solution at 6 and 12 h, and the mixture was then incubated for 4 h in a CO_2_ incubator. Colorimetric changes were quantified at 450 nm using an ELISA reader (Multiskan FC, Thermo FC). Cytotoxicity was evaluated in terms of the inhibition ratio: inhibition ratio = (*OD_E+T_* – *OD*_sample_)/(*OD_E+T_* – *OD*_medium only_), where *OD*, *E* and *T* represent the OD, effector cell and target cells, respectively (target cells include PC3 cells, C4‐2 cells and primary cultured PCa cells from one patient with CRPC).

### 2.11 Degranulation assay

After coincubation with C4‐2 cells (E/T = 1:1) for 20 h, CAR NK‐92 cells were collected and treated with either atezolizumab (20 μg/ml) or nivolumab (20 μg/ml) with Golgi stop protein transport inhibitor (BD Bioscience) for 2 h. Cells were then processed completely according to our previous methods.^21^

### 2.12 Preparation of CD8^+^ T cells cocultured with DCs and C4‐2 cells

Peripheral blood mononuclear cells (PBMCs) were isolated from donated blood samples of normal healthy subjects. Blood was layered on Ficoll‐Paque (TBD Science) and centrifuged at 460 × g for 40 min, washed twice with saline and cultured in DC‐adherent medium composed of X‐VIVO (Lonza) and 5% FBS (Gibco). After 1 h of incubation, adherent PBMCs (monocytes) were collected for DC culture, and suspended PBMCs were collected for CD8^+^ T‐cell culture (Day 0). Adherent monocytes were cultured in a DC growth medium composed of X‐VIVO (Lonza), 5% FBS and 2 U/ml DC culture factors (Novoprotein) in a 37°C CO_2_ incubator, followed by a half replacement of the DC growth medium on Day 3, the addition of DC maturation factors (Novoprotein; final concentration, 2 U/ml) on Day 6 and final collection on Day 8. For CD8^+^ T‐cell culture, the density of suspended PBMCs was adjusted to 1.5 × 10^6^/ml, and the cells were cultured in T‐cell activated medium containing KBM551 (Corning), 3% FBS, 50‐ng/ml anti‐CD3 antibody (Beijing T&L Biological Technology), 1000 U/ml IFN‐γ (Beijing T&L Biological Technology), 100 U/ml interleukin (IL)‐1α (Beijing T&L Biological Technology) and 500 U/ml IL‐2 (SL Pharm). A human CD8^+^ T‐cell isolation kit (Miltenyi Biotec) was used for CD8^+^ T‐cell isolation, which was then cultivated in T‐cell proliferation medium containing KBM 551, 3% FBS and 500 U/ml IL‐2 on Days 3, 4 and 6. On Day 8, approximately 4 × 10^7^ DCs and 1 × 10^8^ CD8^+^ T cells were harvested, centrifuged and then mixed in 150 ml of T‐cell proliferation medium. The mixture of DC‐CD8^+^ T cells was then transferred to a T75 culture flask containing 1 × 10^7^ well‐prepared adherent C4‐2 cells and cocultured in a CO_2_ incubator. After 24 h, the suspended DC‐CD8^+^ T mixture was harvested and selected for CD8^+^ T cells by Miltenyi beads as mentioned above. The selected CD8^+^ T cells were labelled ‘cocultured CD8^+^ T cells’. We also collected CD8^+^ T cells after coculturing with DCs but without C4‐2 cells. The analysis of PBMC phenotypes before and after anti‐CD3 antibody stimulation and cocultured CD8^+^ T cells was performed using flow cytometry, and the cells were stained with the following antibodies: CD3‐APC, CD4‐FITC and CD8‐PE (FITC; BD Bioscience).

### 2.13 Validation of the specific antitumour function of cocultured CD8^+^ T cells in vitro and in vivo

The cytotoxicity of cocultured CD8^+^ T cells against C4‐2 cells in vitro was measured using a CCK‐8 kit, using the PC3 cell line as a control. Cytotoxicity was gauged by the killing rate, which was calculated using the following equation: killing rate = (*OD_T_* − *OD*_sample_/ OD_T_ − OD_medium only_) × 100%, where *OD* and *T* represent the OD and target cells, respectively (target cells including PC3 and C4‐2 cells). For in vivo validation, 2 × 10^6^ C4‐2^GFP^ cells were suspended in 200 μl PBS, followed by subcutaneous injection into the upper abdomen of 5‐week‐old male Nonobese diabetic Severe combined immunodeficiency (NOD/SCID) mice. On Day 5 after injection, the animals were randomly separated into three groups: (1) control, (2) CD8^+^ T cells and (3) cocultured CD8^+^ T cells. PBS (200 μl) was administered via the tail vein in mice of the control group, while mice in the other groups received 1 × 10^7^ cocultured CD8^+^ T or CD8^+^ T cells suspended in PBS (200 μl) in the same way. A calliper was used to measure the longest (L) and shortest (W) diameters of the tumours, and the volumes were calculated using the formula: Volume = L × W^2^/2. BLI was also applied to evaluate the tumour sizes as previously described. In addition, the survival time of the mice was monitored in groups.

### 2.14 Mice and procedures

Male NOD/SCID mice aged 5 weeks were purchased from the Beijing Vital River Laboratory Animal Technology Company, raised and treated according to protocols approved by the Ethical Committee of the National Cancer Center. Mice were inoculated with C4‐2 cells as previously described. Treatment started on Day 7 when the tumour volume reached 100–200 mm^3^, and C4‐2‐inoculated mice were allocated randomly into seven groups: (1) control, (2) CAR NK‐92, (3) CAR NK‐92+nivolumab, (4) CAR NK‐92+atezolizumab, (5) CAR NK‐92+cocultured CD8^+^ T cells, (6) CAR NK‐92+nivolumab+cocultured CD8^+^ T cells and (7) CAR NK‐92+atezolizumab+cocultured CD8^+^ T cells. The PD‐L1 antibody atezolizumab (GlpBio, GC32704; 20 mg/kg), PD‐1 antibody nivolumab (GlpBio, GC34218; 10 mg/kg) or control PBS was administered on Days 7, 9, 11, 13 and 15 after tumour inoculation via the tail vein, while CAR NK‐92 cell treatment was administered by injecting 5 × 10^6^ CAR NK‐92 cells intravenously on Days 8, 10, 12, 14 and 16, 1 day after PD‐L1/PD‐1 antibody therapy. Cocultured CD8^+^ T cells were intravenously injected on Day 17 after CAR NK‐92 treatment with or without PD‐L1/PD‐1 antibody. Tumour volumes were calculated as described on Days 7, 10, 14, 16, 18 and 20. Tumour sizes were also evaluated by BLI on Days 7, 14 and 21 after tumour cell implantation, and BLI was measured and expressed as radiance (p/s/cm2/sr). All mice were killed on Day 21, and the implanted tumours were collected, photographed, weighed and collected for histological examination. In addition to the tumour progression evaluated by BLI and callipers, we set mouse satellite groups to monitor survival time (*n* = 4 in each group).

### 2.15 Histology and quantification of necrosis

This part of the work was performed according to our previous methods.^21^

### 2.16 Primary culture of PCa cells from a patient with CRPC

PCa tissues were collected from one patient with CRPC who underwent a radical prostatectomy in our hospital. Personal information was not used in the text or illustrative materials of this research.

Patient baseline information: A 67‐year‐old man with a chief complaint of pelvic and leg pain for 9 months and no other significant medical history of concomitant illnesses consulted a doctor at a local hospital. Digital rectal examination detected a 2 × 2 cm nodular mass. The prostate‐specific antigen (PSA) value was 18.18 ng/ml in July 2020. Choline positron emission tomography/computed tomography (PET/CT) showed high tracer concentrations in the prostate, iliac and pubic bone regions, and a bone scan showed multiple bone lesions (in the right iliac bone, bilateral pubic bone, bilateral ramus of the ischium and sacrum). Moreover, magnetic resonance imaging (MRI) confirmed the presence of PCa and multiple lesions in the pelvis. In August 2020, he was diagnosed with adenocarcinoma with a Gleason score of 4 + 4 by transrectal ultrasound‐guided biopsy at our hospital and was staged as cT3aN0M1b. He received ADT, which included abiraterone prednisone/prednisolone in combination with goserelin acetate therapy (a gonadotropin‐releasing hormone agonist 3.6 mg ih once every 4 weeks). After 3 months of ADT, the patient responded well, and his PSA level dropped to 0.279 ng/ml in November 2020. However, his PSA levels began to increase over the nadir from May 2021 under a castration level of testosterone (the castration level was defined as < 50 ng/ml or 1.7 nmol/L). Preoperative pelvic MRI demonstrated a 1.5 × 1.2 cm lesion in the peripheral zone of the left lobe, which was considered a tumour residue after ADT. No signs of seminal vesicle invasion or lymph node enlargement were observed. The patient successfully underwent robot‐assisted radical prostatectomy (RARP) and bilateral pelvic lymphadenectomy under general anaesthesia on 26 May 2021. The postoperative histopathological report revealed a Gleason Grade 9 (4 + 5) left lobe adenocarcinoma with extraprostatic invasion, positive surgical margins at the apex, tumour emboli, neural invasion and positive lymph nodes (left 2/7, right 0/4). The pathological stage of the tumour was ypT3aN1. IHC indicated AE1/AE3 (3+), P504S (2+), PSA (2+), CD56 (1+), Syno (focal point+), ChrA (focal point+), P53 (+,20%) and Ki‐67 (+ dense area 5%). Part of the fresh specimen was sent to the laboratory for primary culture of PCa cells within 2 h of removal. The procedure was as follows: the CRPC tissue was rinsed and cut into 3‐mm‐thick pieces. Then, these pieces were digested for 2 h in alpha‐MEM (Corning) with 0.1% collagenase I (Sigma) at 37°C. A 10‐cm cell culture dish was precoated with FBS (Gibco), and the isolated pieces of tissue were planted on the dish without FBS in a CO_2_ incubator for 24 h. Then, 10 ml alpha‐MEM supplemented with 10% FBS was added to the dish for further cell cultures.

### 2.17 Statistical analysis

All experiments were performed in triplicate, and the data are shown as the mean ± standard deviation. All statistical analyses were conducted using SPSS 22.0 software (SPSS). Comparisons of multiple groups were conducted using one‐way analysis of variance followed by Tukey's post hoc test, and the independent two groups were compared using Student's *t*‐test. For data that were not normally distributed, the Kruskal–Wallis test and Mann–Whitney *U* test were used for comparisons of multiple and two groups, respectively. The Kaplan–Meier method was used to plot survival curves for each group of mice, and the log‐rank test was performed to compare differences between groups. Two‐sided *p* < .05 was considered statistically significant.

## 3 RESULTS

### 3.1 Expression of PD‐L1 on C4‐2 cells increased after coculture with CAR NK‐92 cells in an IFN‐γ‐dependent manner

**FIGURE 1:**
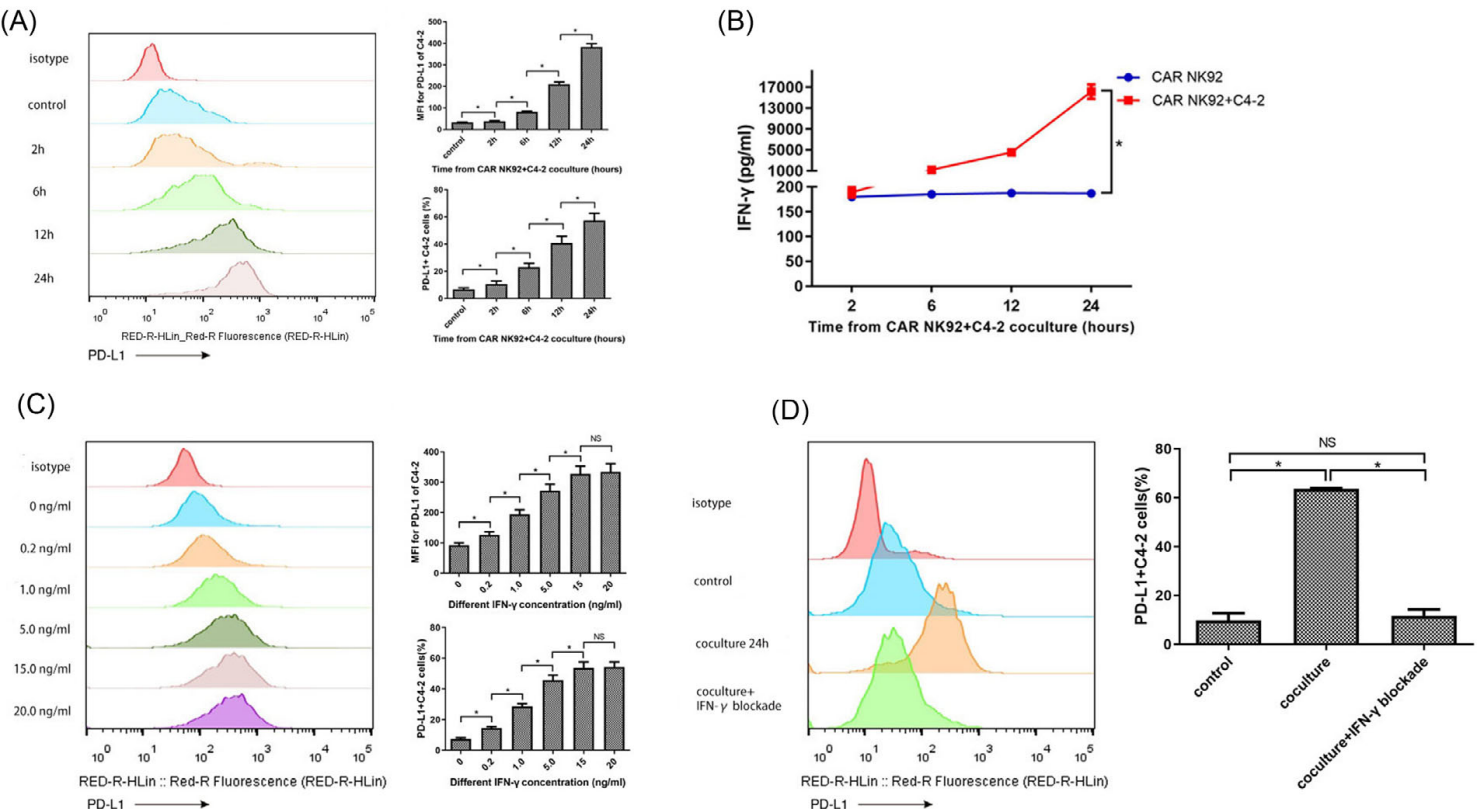
Upregulation of programmed cell death‐ligand 1 (PD‐L1) expression on C4‐2 cells cocultured with chimeric antigen receptor NK‐92 (CAR NK‐92) cells was dependent on interferon‐γ (IFN‐γ). (A) C4‐2 cells were cocultured with CAR NK‐92 cells for 2, 6, 12 and 24 h, and the expression of PD‐L1 on C4‐2 cells was detected by flow cytometry; the representative images and summary data (*n* = 3) are shown. The median fluorescence intensity (MFI) for PD‐L1 and the percentages of PD‐L1+ C4‐2 cells were significantly increased in a time‐dependent manner. (B) IFN‐γ concentrations in the culture supernatant of CAR NK‐92 cells cocultured with or without C4‐2 cells were measured by ELISA at 2, 6, 12 and 24 h. The experiments were repeated three times. (C) Representative flow cytometry plots and summary data (*n* = 3) of PD‐L1 expression on C4‐2 cells in response to stimulation with different concentrations of IFN‐γ. The MFI for PD‐L1 and PD‐L1+ percentages increased in a concentration‐dependent manner. (D) Representative flow cytometry images and summary data (*n* = 3) of PD‐L1 expression on C4‐2 cells cocultured with CAR NK‐92 cells for 24 h with or without an IFN‐γ blocker. IFN‐γ blocker completely reversed PD‐L1 upregulation in C4‐2 cells cocultured with CAR NK‐92 cells. Bars represent the means ± standard deviation (SD), one‐way analysis of variance (ANOVA) followed by a Tukey post hoc test was used for multiple group comparisons (A, C, D), and Student's *t*‐test was used for two‐group comparisons (B). *p < .05; Median fluorescence intensity; NS, not significant

The baseline expression of PD‐L1 on C4‐2 cells was approximately 9.3% and was significantly upregulated in a time‐dependently after coculturing with CAR NK‐92 cells (Figure 1A). After 24 h of coculture, PD‐L1 expression on C4‐2 cells increased to 58.4%. In addition, we tested IFN‐γ levels in the supernatant of the coculture at 2, 6, 12 and 24 h and observed that IFN‐γ levels markedly increased with time, from 188.73 to 16105.3 pg/ml (Figure 1B). To test whether PD‐L1 upregulation was dependent on IFN‐γ, we stimulated C4‐2 cells with different IFN‐γ concentrations found in the supernatant and quantified PD‐L1 expression. The results demonstrated that IFN‐γ concentration and PD‐L1 expression have a notable positive correlation, which was in line with the coculture data (Figure 1C). Furthermore, we added an IFN‐γ blocker at the beginning of coculturing and found that complete blockade of IFN‐γ reversed PD‐L1 upregulation in C4‐2 cells cocultured with CAR NK‐92 cells for 24 h (Figure 1D). Collectively, PD‐L1 on the surface of C4‐2 cells was upregulated when C4‐2 cells encountered activated CAR NK‐92 cells, which was dependent on IFN‐γ secreted by CAR NK‐92 cells.

### 3.2 Contact‐dependent upregulation of PD‐L1 on CAR NK‐92 cells increased after coculture with C4‐2 cells

**FIGURE 2:**
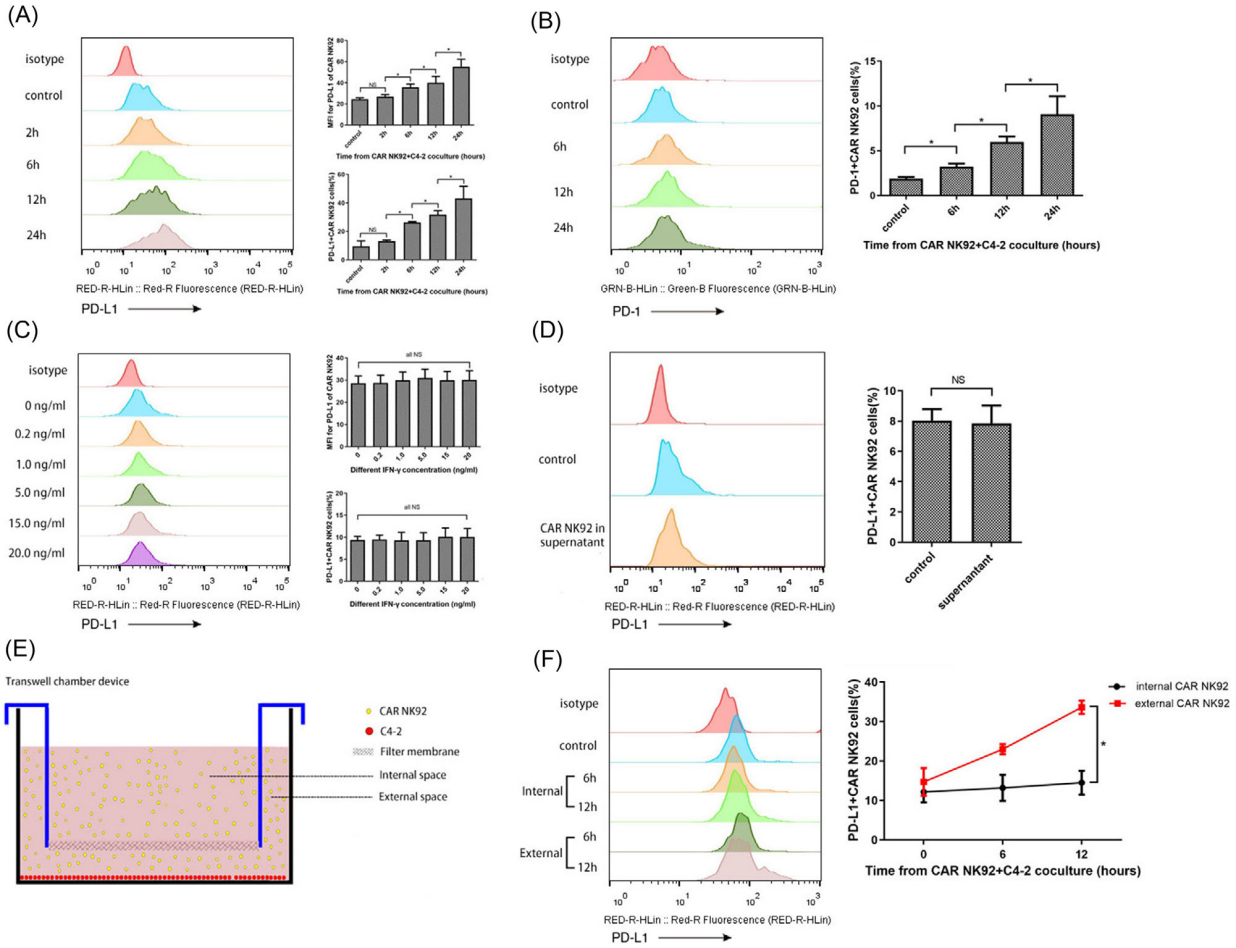
Upregulation of PD‐L1 expression on CAR NK‐92 cells cocultured with C4‐2 cells was dependent on direct cell contact. (A) Representative flow cytometry images and summary data (*n* = 3) showing the expression of PD‐L1 on CAR NK‐92 cells cocultured with C4‐2 cells for 2, 6, 12 and 24 h. The MFI for PD‐L1 and PD‐L1+ percentages of CAR NK‐92 cells were significantly increased in a time‐dependent manner. (B) Representative flow cytometry images and summary data (*n* = 3) showing the expression of PD‐1 on CAR NK‐92 cells cocultured with C4‐2 cells for 6, 12 and 24 h. The percentages of PD‐1+ CAR NK‐92 cells were significantly upregulated in a time‐dependent manner. (C) PD‐L1 expression on CAR NK‐92 cells stimulated with different concentrations of IFN‐γ (*n* = 3). There was no significant change in MFI for PD‐L1 or PD‐L1+ percentages of CAR NK‐92 cells after IFN‐γ stimulation. (D) Representative flow cytometry images and summary data (*n* = 3) showing the expression of PD‐L1 on CAR NK‐92 cells incubated with normal medium or supernatants acquired from coculture medium for 12 h. There was no significant difference in PD‐L1+ percentages of CAR NK‐92 cells cultured in the two culture mediums. (E) Schematic diagram of the trans‐well chamber device. C4‐2 cells were seeded at the bottom, and CAR NK‐92 cells were cultured in the full medium of the device. The internal and external space was separated by a filter membrane, which allowed the cytokines to pass through but blocked the exchange of CAR NK‐92 cells. (F) PD‐L1 expression on CAR NK‐92 cells cultured in internal and external spaces for 6 and 12 h (*n* = 3). The PD‐L1+ percentages of CAR NK‐92 cells incubated in an external space were significantly increased at 6 and 12 h, while the PD‐L1+ percentages of CAR NK‐92 cells incubated in a trans‐well chamber did not show any significant changes. Bars represent the means ± SD, ANOVA followed by a Tukey post hoc test was used for multiple group comparisons (A, B, C), and Student's *t*‐test was used for two‐group comparisons (D, F). *p < .05; NS, not significant

We measured PD‐L1 expression at different time points on CAR NK‐92 cells cocultured with C4‐2 cells and found that PD‐L1 expression was upregulated markedly in a time‐dependent manner (Figure 2A). Interestingly, we observed that PD‐1 expression on CAR NK‐92 cells also increased when cocultured with C4‐2 cells (Figure 2B). We then checked whether PD‐L1 upregulation was also dependent on IFN‐γ as with C4‐2 cells. We stimulated CAR NK‐92 cells with IFN‐γ using the same method and found that IFN‐γ did not alter the expression level of PD‐L1 in CAR NK‐92 cells (Figure 2C), which was unlike its role in modulating PD‐L1 on C4‐2. To explore the mechanism of C4‐2‐induced upregulation of PD‐L1 in CAR NK‐92 cells, we used two methods to test whether direct cell interaction was necessary. First, CAR NK‐92 cells were cultured in the supernatants from C4‐2 cells alone or those from C4‐2 cells incubated with CAR NK‐92 cells. The data showed that the cocultured supernatants could not induce PD‐L1 upregulation on CAR NK‐92 cells (Figure 2D). Second, a trans‐well chamber device was used to simultaneously test PD‐L1 expression on CAR NK‐92 cells incubated both in a trans‐well chamber and an external space (Figure 2E). If direct cell contact was not necessary, then cytokines secreted by external CAR NK‐92 cells with C4‐2 cell contact at the bottom would pass through the membrane filter and influence PD‐L1 expression on CAR NK‐92 cells incubated in the trans‐well chamber. We found that the expression of PD‐L1 in CAR NK‐92 cells incubated in the external space was upregulated at 6 and 12 h, while its counterpart incubated in a trans‐well chamber did not show any significant changes in PD‐L1 expression (Figure 2F). Collectively, the direct contact between CAR NK‐92 and tumour cells, rather than cytokines, was necessary to upregulate PD‐L1 expression on effector cells.

### 3.3 The NKG2D/PI3K/AKT/mTOR signalling pathway regulated the expression of PD‐L1 in CAR NK‐92 cells

**FIGURE 3:**
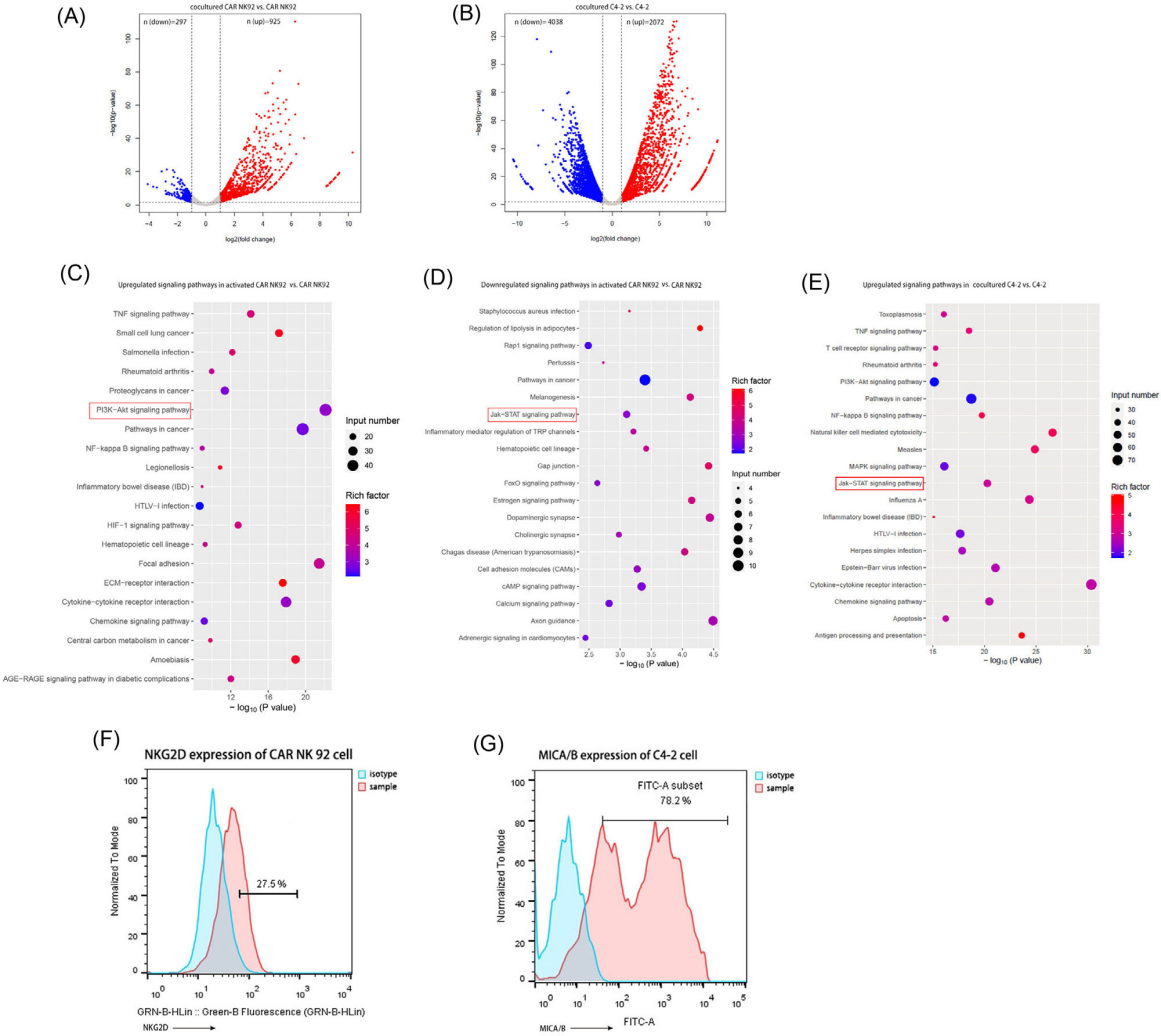
Transcriptional analysis of CAR NK‐92 and C4‐2 cells after coculture. (A) Volcano plots of the dysregulated genes between cocultured and uncocultured CAR NK 92 cells. (B) Volcano plots of the dysregulated genes between cocultured and uncocultured C4‐2 cells. In (A) and (B), genes differentially expressed with a fold change greater than 2.0 and *p* < .05 are marked in colour. The *p*‐values were calculated using a two‐sided unpaired Student's *t*‐test. (C) Kyoto Encyclopedia of Genes and Genomes (KEGG) pathway enrichment analysis of upregulated genes in CAR NK‐92 cells following incubation with C4‐2 cells. (D) KEGG pathway enrichment analysis of downregulated genes in CAR NK‐92 cells following incubation with C4‐2 cells. (E) KEGG pathway enrichment analysis of upregulated genes in C4‐2 cells following coculture with CAR NK‐92 cells. In (C, D, E,) the colour of the dots represents the rich factor, and the size represents the input number of each KEGG term. The horizontal axis indicates the significance of enrichment. The vertical axis indicates the enriched KEGG pathway (20 most enriched terms). (F) Representative flow cytometry plots and data (*n* = 3) of NKG2D expression on CAR NK‐92 cells. (G) Representative flow cytometry plots and data (*n* = 3) of MICA/B expression in C4‐2 cells.

**FIGURE 4:**
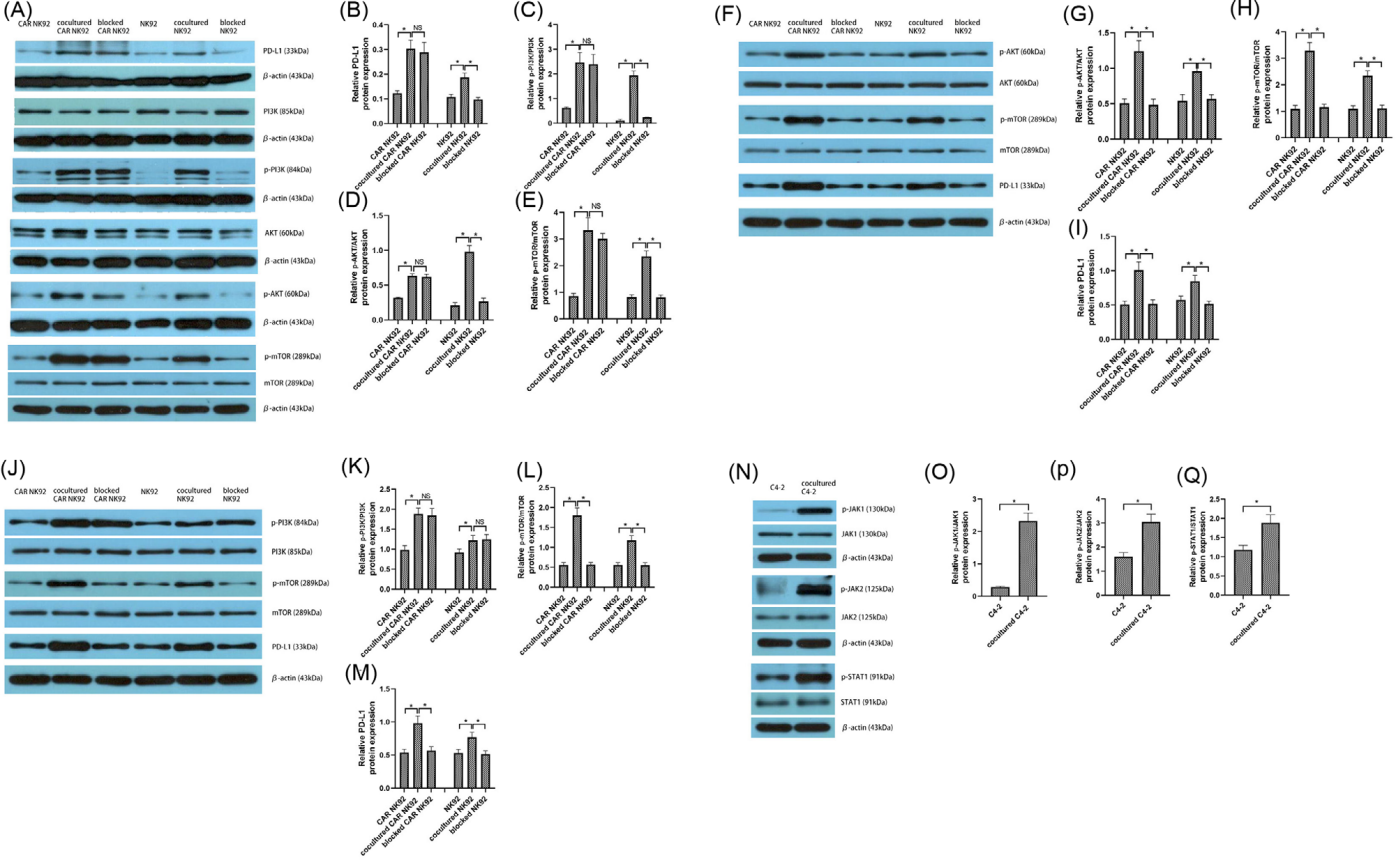
Western blotting analysis of signalling pathway molecules involved in PD‐L1 expression on NK‐92, CAR NK‐92 and C4‐2 cells when cocultured with each other. (A–E) Representative and summary data (*n* = 3) of PD‐L1 (A, B), p‐PI3K (A, C), p‐AKT (A, D) and phosphorylated mammalian target of rapamycin (p‐mTOR) (A, E) protein levels in a panel of CAR NK‐92 cells, cocultured CAR NK‐92 cells, blocked CAR NK‐92 cells (treated with NKG2D blocker), NK‐92 cells, cocultured NK‐92 cells and blocked NK‐92 cells (treated with NKG2D blocker). The NKG2D blocker was added to effector cells and incubated for 1 h at 37°C prior to coculture with C4‐2 cells. (F–I) Representative and summary data (*n* = 3) of p‐AKT (F, G), p‐mTOR (F, H) and PD‐L1 (F, I) protein levels in a panel of CAR NK‐92 cells, cocultured CAR NK‐92 cells, blocked CAR NK‐92 cells (treated with wortmannin), NK‐92 cells, cocultured NK‐92 cells and blocked NK‐92 cells (treated with wortmannin). Effector cells were cocultured with C4‐2 cells in the absence or presence of the PI3K‐specific inhibitor wortmannin (10 μmol/L). (J–M) Representative and summary data (*n* = 3) of the protein levels of p‐PI3K (J, K), p‐mTOR (J, L) and PD‐L1 (J, M) in a panel of CAR NK‐92 cells, cocultured CAR NK‐92 cells, blocked CAR NK‐92 cells (treated with afuresertib), NK‐92 cells, cocultured NK‐92 cells and blocked NK‐92 cells (treated with afuresertib). Effector cells were incubated with C4‐2 cells in the absence or presence of the AKT inhibitor afuresertib (10 μmol/L). (N–Q) Representative and summary data (*n* = 3) of JAK/STAT pathway intermediaries, including p‐JAK1 (N, O), p‐JAK2 (N, P) and p‐STAT1 (N, Q) in C4‐2 cells cocultured with CAR NK‐92 cells compared with C4‐2 cells without any process. CAR NK‐92 or NK‐92 cells were cocultured with C4‐2 cells for 24 h and then harvested for western blotting. Bars represent the means ± SD, ANOVA followed by a Tukey post hoc test was used for multiple group comparisons (B–E, G–I, K–M), and Student's *t‐*test was used for two‐group comparisons (O–Q). *p < .05; NS, not significant

To investigate the exact mechanisms of PD‐L1 upregulation in CAR NK‐92 cells, we performed RNA sequencing to profile the genes expressed in both activated and inactivated CAR NK‐92 cells. RNA sequencing of C4‐2 cells was also performed, regardless of whether they were cocultured with CAR NK‐92 cells. There were 925 upregulated genes and 297 downregulated genes when comparing activated CAR NK‐92 cells with their inactivated counterparts (Figure 3A) and 2072 upregulated genes and 4038 downregulated genes in cocultured C4‐2 cells, compared to their uncocultured counterparts (Figure 3B). KEGG analysis revealed significantly upregulated and downregulated signalling pathways in activated CAR NK‐92 cells (Figure 3C,D). Interestingly, we found that JAK‐STAT, the well‐known IFN‐γ‐mediated signalling pathway responsible for regulating tumour PD‐L1 expression, was downregulated, whereas the PI3K/AKT, TNF, HIF‐1 and nuclear factor (NF)‐κB signalling pathways were remarkably enriched. After a comprehensive analysis of all types of regulation in PD‐L1 expression^22^ and all NK‐cell signalling pathways,^23^ we noticed that the PI3K/AKT pathway was the only pathway responsible for the upregulation of PD‐L1 in activated CAR NK‐92 cells. In addition, KEGG analysis showed that the JAK‐STAT signalling pathway was upregulated in C4‐2 cells cocultured with CAR NK‐92 cells, compared to that in C4‐2 cells alone (Figure 3E). To test our hypothesis that the NKG2D/PI3K/AKT/mTOR signalling pathway regulates the expression of PD‐L1 in effector cells, we first measured the expression of NKG2D and MICA/MICB and observed that the expression of NKG2D on CAR NK‐92 cells was 27.5% (Figure 3F) and the expression of MICA/MICB on C4‐2 cells was 78.2% (Figure 3G). Next, we incubated effector cells with C4‐2 cells in the presence of an NKG2D blocker and then measured the expression of PI3K/AKT/mTOR‐related activated molecules and PD‐L1 in effector cells. Treatment with an NKG2D blocker significantly reduced the expression of PD‐L1, p‐PI3K, p‐AKT and p‐mTOR in NK‐92 cells. However, CAR NK‐92 expression of these signalling proteins was not inhibited by the NKG2D blocker (Figure 4A–E). To further support our hypothesis, we incubated CAR NK‐92 or NK‐92 cells with C4‐2 cells in the presence of the PI3K inhibitor wortmannin, measured the expression of downstream molecules and found that wortmannin significantly reduced p‐AKT, p‐mTOR and PD‐L1 expression in both effector cell lines (Figure 4F–I). Downstream of the PI3K cascade is AKT; treatment with the global AKT inhibitor afuresertib evidently reduced p‐mTOR and PD‐L1 expression in both effector cells when incubated with C4‐2 cells, while it did not interfere with upstream PI3K activation (Figure 4J–M). In addition, the western blotting results showed that the main JAK‐STAT pathway‐related proteins, including JAK1/2 and STAT1, were activated in C4‐2 cells cocultured with CAR NK‐92 cells (Figure 4N–Q). In summary, these data suggest that the NKG2D/PI3K/AKT/mTOR pathway mediates PD‐L1 upregulation in activated CAR NK‐92 cells.

### 3.4 Atezolizumab enhanced the cytotoxicity of CAR NK‐92 cells in vitro

**FIGURE 5:**
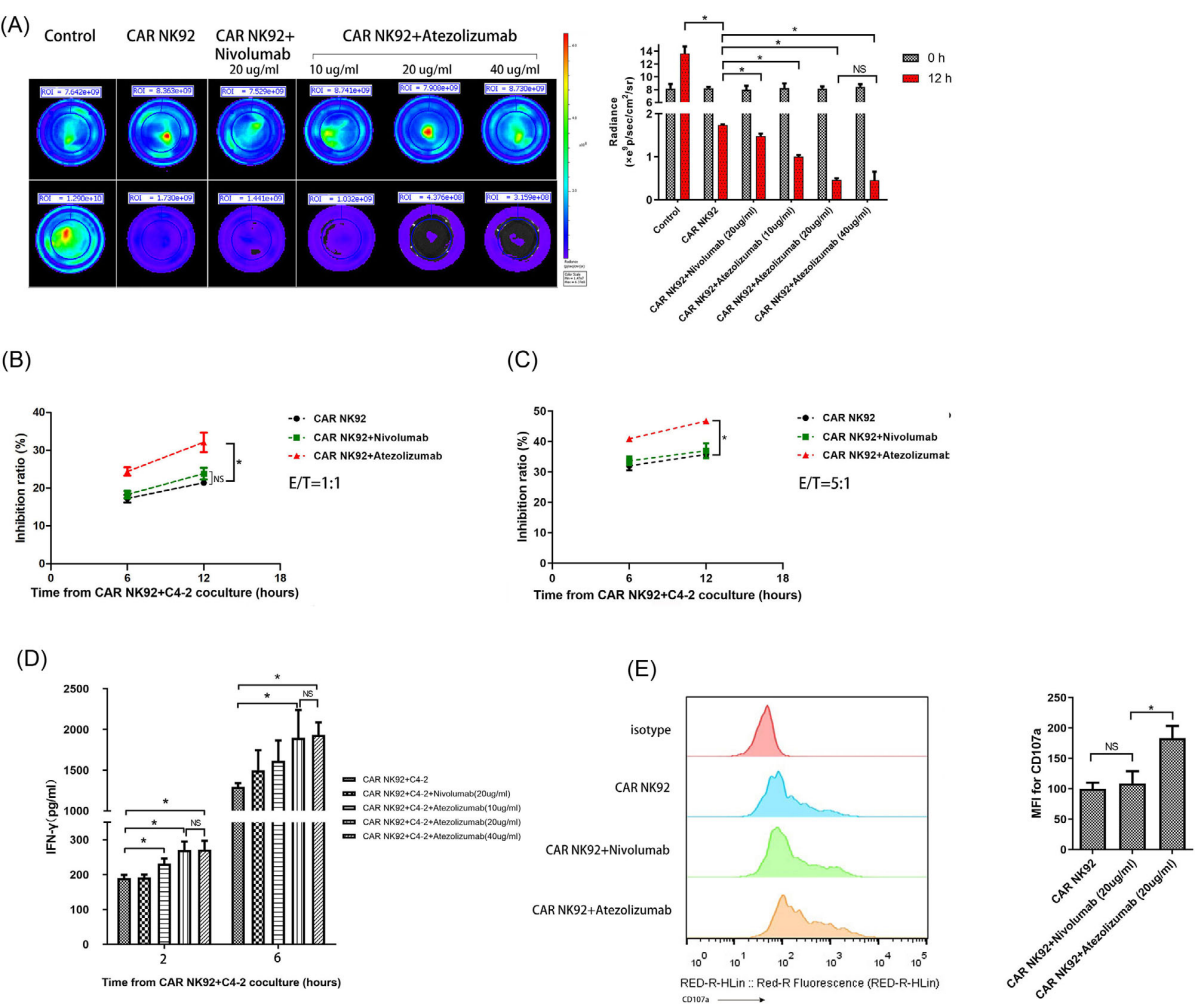
Detection of the cytotoxic activity of CAR NK‐92 cells treated with atezolizumab or nivolumab. (A) Representative BLI of C4‐2^GFP^ cells in the control and treatment groups, including the CAR NK‐92, CAR NK‐92+nivolumab (20 μg/ml) and CAR NK‐92+atezolizumab (10, 20, 40 μg/ml) groups (*n* = 3). (B, C) The inhibition ratios of CAR NK‐92, CAR NK‐92+nivolumab (20 μg/ml), CAR NK‐92 + atezolizumab (20 μg/ml) treatment measured using the Cell Counting Kit‐8 (CCK‐8) assay (*n* = 3). E/T = 1:1, 5:1, respectively. (D) IFN‐γ levels in the culture supernatant of CAR NK‐92 cells cocultured with C4‐2 cells in the control and treatment groups, including CAR NK‐92, CAR NK‐92 + nivolumab (20 μg/ml) and CAR NK‐92+atezolizumab (10, 20, 40 μg/ml), measured by ELISA at 2 and 6 h (*n* = 3). (E) Representative flow cytometry plots and summary data (*n* = 3) showing CD107a expression in CAR NK‐92 cells cocultured with C4‐2 cells in the presence of atezolizumab or nivolumab. Degranulation of CAR NK‐92 cells was induced upon interaction with C4‐2 cells at a 1:1 ratio for 20 h at 37°C, and then CAR NK‐92 cells were collected and treated with atezolizumab (20 μg/ml) or nivolumab (20 μg/ml), followed by flow cytometry measurement. Data expressed as the means ± SD were plotted, and ANOVA followed by a Tukey post hoc test was used to compare three or more groups (A–E). *p < .05; BLI, bioluminescent intensity; E/T, effector‐to‐target ratio; NS, not significant

To further investigate the influence of PD‐L1 on CAR NK‐92 cells, we used atezolizumab, an Food and Drug Administration (FDA)‐approved humanised immunoglobulin (Ig) G1 mAb against PD‐L1,^24^, ^25^ to treat CAR NK‐92 cells cocultured with C4‐2 cells. For comparison, we used nivolumab, an FDA‐approved humanised IgG4 mAb against PD‐1, to treat head and neck cancers.^26^ Our results demonstrated that atezolizumab enhanced CAR NK‐92 cell cytotoxicity against PCa cells dose‐dependently as reflected by the negative correlation between the atezolizumab concentration and the BLI of C4‐2 cells (Figure 5A). However, when the dosage was increased from 20 to 40 μg/ml, the cytotoxicity reached a plateau, which may be the result of saturation of atezolizumab binding on PD‐L1. Interestingly, nivolumab also enhanced CAR NK‐92 cell cytotoxicity against C4‐2 cells. Overall, the CCK‐8 assay data supported the BLI results and showed that the inhibition ratio of CAR NK‐92 cells combined with atezolizumab therapy was evidently higher than that of CAR NK‐92 cells alone (Figure 5B,C). Although not as evident as the BLI results, the CCK‐8 assay indicated that nivolumab marginally increased the inhibition ratio of CAR NK‐92 cells. We conclude that the difference in BLI or inhibition ratio (atezolizumab minus nivolumab) indicates the pure contribution of atezolizumab independent of the PD‐L1/PD‐1 axis in enhancing CAR NK‐92 cell cytotoxicity. In addition, we observed that atezolizumab obviously enhanced CAR NK‐92 production of IFN‐γ after coculturing with C4‐2 cells for 2 and 6 h (Figure 5D). We also observed that C4‐2‐cocultured CAR NK‐92 cells expressed more CD107a in the presence of atezolizumab than in the absence of any ICI or in the presence of nivolumab (Figure 5E).

### 3.5 Atezolizumab augmented the antitumour effect of CAR NK‐92 cells on C4‐2 xenografts in vivo

**FIGURE 6:**
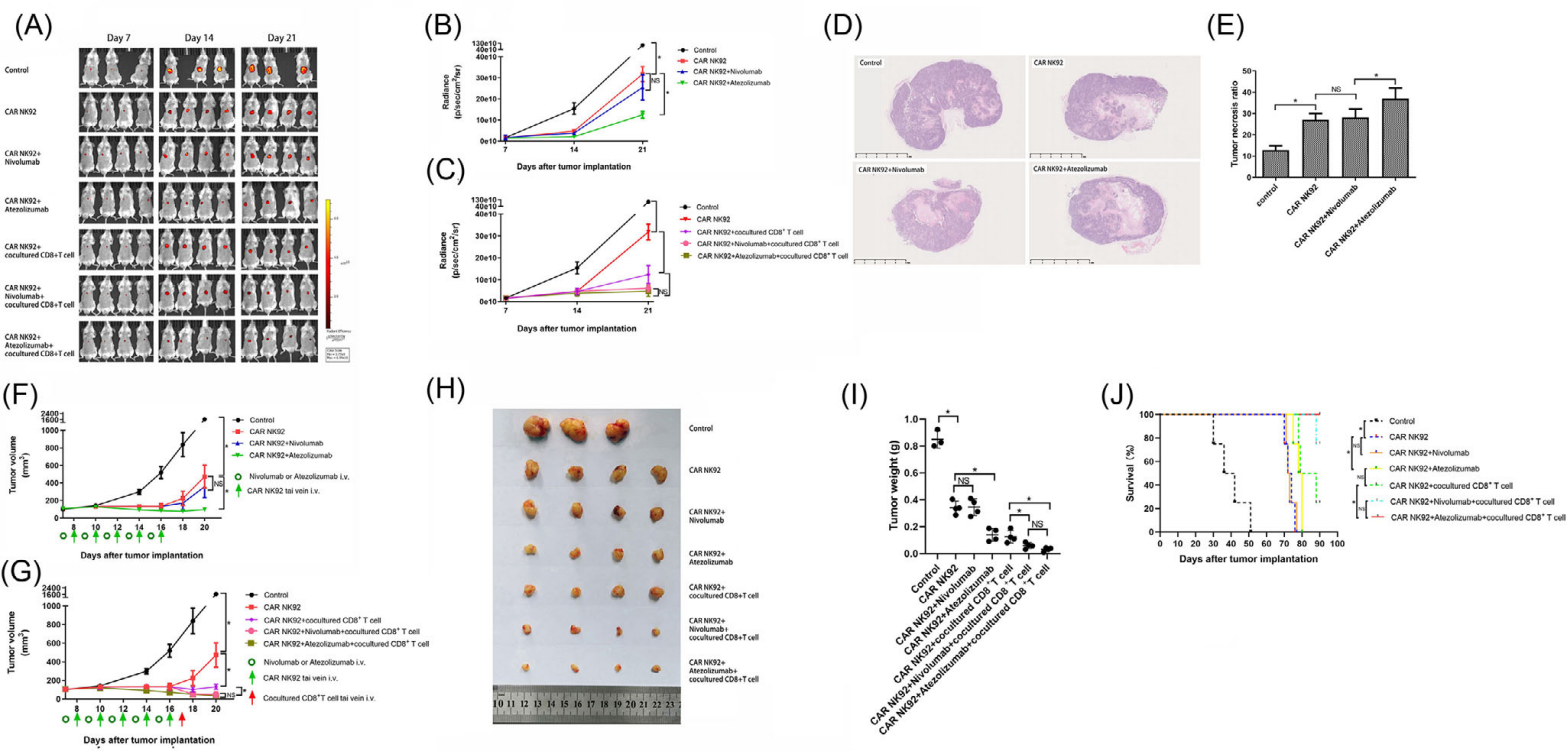
Antitumour effect of CAR NK‐92 cells against C4‐2 cells in combination with atezolizumab or nivolumab in the presence or absence of CD8^+^ T cells in vivo. (A) BLI measurements of the tumours arising from C4‐2^GFP^ cells implanted in mice were performed in the control and treatment groups, including CAR NK‐92, CAR NK‐92+nivolumab, CAR NK‐92+atezolizumab, CAR NK‐92+cocultured CD8^+^ T cells, CAR NK‐92+nivolumab + cocultured CD8^+^ T cells and CAR NK‐92+atezolizumab + cocultured CD8^+^ T‐cell groups on Days 7, 14 and 21 (n = 3–4). (B) Quantitative BLIs of tumour activity in the control and treatment groups, including the CAR NK‐92, CAR NK‐92 + nivolumab and CAR NK‐92 + atezolizumab groups, on Days 7, 14 and 21 (*n* = 3–4). (C) Quantitative BLIs of tumour activity in the control and treatment groups, including CAR NK‐92, CAR NK‐92 + cocultured CD8^+^ T cells, CAR NK‐92+nivolumab + cocultured CD8^+^ T cells and CAR NK‐92 + atezolizumab + cocultured CD8^+^ T cells, on Days 7, 14 and 21 (*n* = 3–4). (D) Representative HE‐stained tumour sections from the control, CAR NK‐92, CAR NK‐92 + nivolumab and CAR NK‐92 + atezolizumab groups (*n* = 3–4). Evident tumour necrosis was observed in the CAR NK‐92 cell treatment group; atezolizumab significantly increased the severity of CAR NK‐92 cell‐induced necrosis. (E) Statistical analyses of the tumour necrosis ratio at Day 21 after implantation (*n* = 3–4). (F) Tumour volumes in the control and treatment groups, including CAR NK‐92, CAR NK‐92 + nivolumab and CAR NK‐92 + atezolizumab groups, were assessed on Days 7, 10, 14, 16, 18 and 20. Tumour volumes were calculated according to the formula L × W^2^/2, where L and W represent the longest and shortest diameters measured by a calliper, respectively (*n* = 3–4). (G) Tumour volumes in the control and treatment groups, including CAR NK‐92, CAR NK‐92 + cocultured CD8^+^ T cell, CAR NK‐92 + nivolumab + cocultured CD8^+^ T cell, CAR NK‐92 + atezolizumab + cocultured CD8^+^ T‐cell groups on Days 7, 10, 14, 16, 18 and 20 (*n* = 3–4). (H) Tumours excised from euthanised mice were photographed, measured and compared in each group (*n* = 3–4). (I) Tumour weights corresponding to each group when harvested on Day 21 (*n* = 3–4). (J) Cumulative Kaplan–Meier survival curves for mice (*n* = 4, log‐rank test). Values are expressed as the means ± SD, and ANOVA followed by a Tukey post hoc test was used for multiple group comparisons (B, C, E, F, G, I). The Kaplan–Meier method was used to estimate survival functions, and the log‐rank test was used for group comparisons (J). *p < .05; BLI, bioluminescent intensity; NS, not significant

In this section, we focused on the antitumour effect of CAR NK‐92 cells combined with atezolizumab without CD8^+^ T cells. BLIs did not exhibit any obvious differences between the groups on Day 7. As predicted from our earlier research in vitro, the BLI on Days 14 and 21 of the CAR NK‐92 cells combined with atezolizumab group was evidently lower than that of the CAR NK‐92 alone group (Figure 6A,B). This finding indicates that in vivo administration of combined CAR NK‐92 cells and atezolizumab had more evident effects on controlling tumour growth than CAR NK‐92 cell monotherapy. In line with the in vivo BLI results, the volume and masses of tumours treated with CAR NK‐92 cells combined with atezolizumab significantly decreased, compared to those of tumours treated with CAR NK‐92 cell monotherapy (Figure 6F,H,I). The hematoxylin and eosin (HE) examination demonstrated that, compared to CAR NK‐92 cell monotherapy, the combined treatment caused an obvious increase in the percentage of necrosis area in the tumour specimen (Figure 6D,E). However, the PD‐1 inhibitor nivolumab did not significantly improve the in vivo antitumour effect of CAR NK‐92 cells regarding BLI, tumour volume, tumour mass or necrosis area (Figure 6A,B,D,E,F,H,I).

### 3.6 Acquisition of cocultured CD8^+^ T cells with specific recognition of C4‐2

**FIGURE 7:**
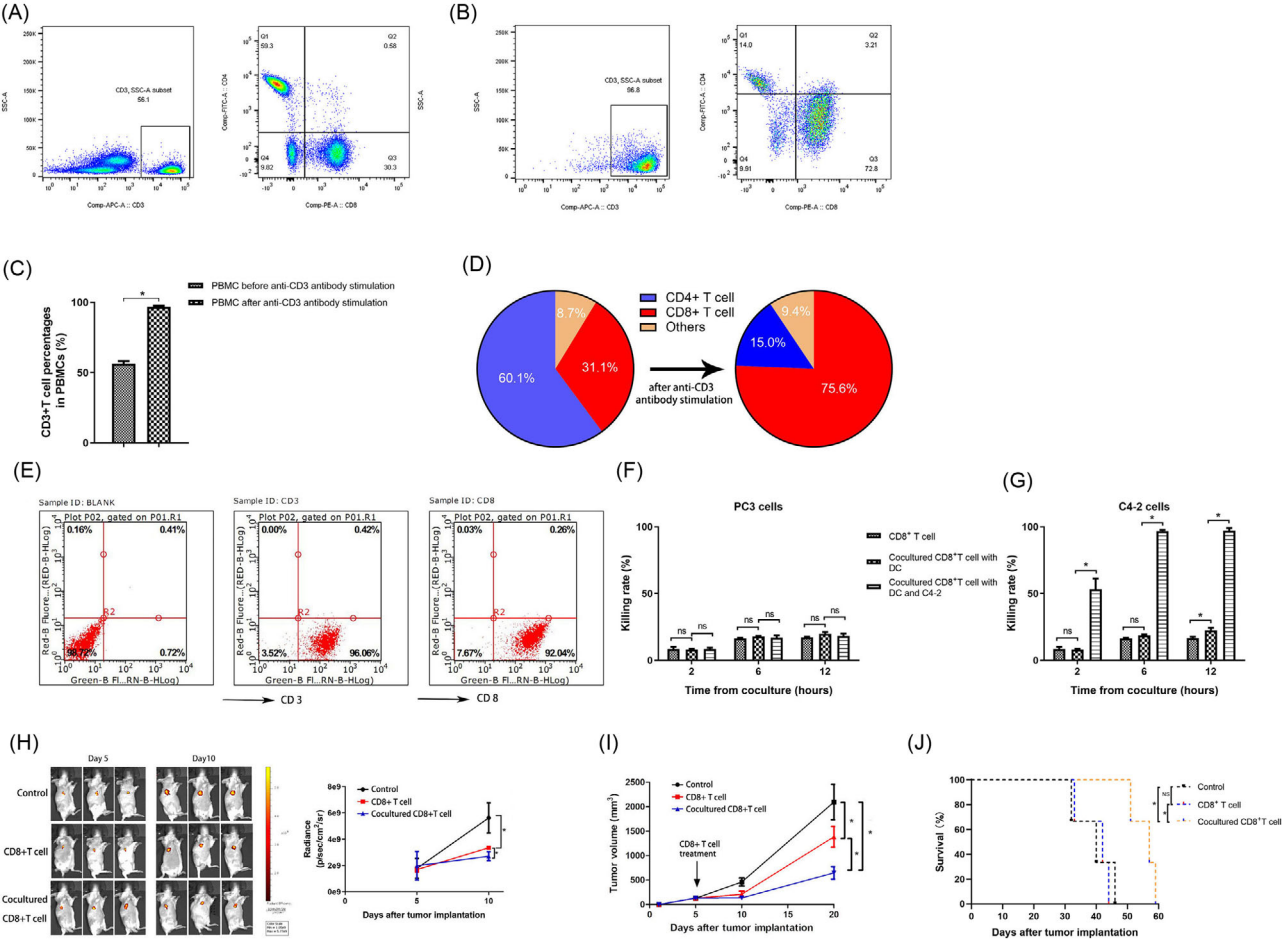
Identification of PBMC constitution in the CD8^+^ T‐cell–cell culture process and functional verification of the cocultured CD8^+^ T cells. (A) Detection of CD3 expression in PBMCs and CD4 and CD8 expression in selected T cells among PBMCs using flow cytometry (*n* = 3) before incubation with anti‐CD3 antibody, IFN‐γ, IL‐1α and IL‐2. (B) Detection of CD3 expression in PBMCs and CD4 and CD8 expression in T cells using flow cytometry after culture with anti‐CD3 antibody and other cytokines, including IFN‐γ, IL‐1α and IL‐2. (**C)** Statistical analysis of the results of CD3+ T‐cell percentages in PBMCs before and after culture with anti‐CD3 antibody and other cytokines (*n* = 3). (D) Statistical analysis of the CD4^+^ and CD8^+^ T‐cell percentages in the selected T cells of PBMCs before and after culture with anti‐CD3 antibody and other cytokines (*n* = 3). (E) Detection of CD3 and CD8 expression in the final cocultured CD8^+^ T cells using flow cytometry (*n* = 3). The cocultured CD8^+^ T cells were acquired after incubation with DCs, CD8^+^ T cells and C4‐2 cells. (F) The cytotoxicity of CD8^+^ T cells, CD8^+^ T cells cocultured with DCs and CD8^+^ T cells cocultured with DCs and C4‐2 cells against PC3 cells was assessed by CCK‐8 assays at the indicated time points. (G) The cytotoxicity of CD8^+^ T cells, CD8^+^ T cells cocultured with DCs and CD8^+^ T cells cocultured with DCs and C4‐2 cells against C4‐2 cells was assessed by CCK‐8 assays at the indicated time points. Data are shown as the mean ± SD of three independent experiments for (F and G). *p < .05; DC, dendritic cell; NS, not significant. (H) BLI measurements of the tumours originating from C4‐2^GFP^ cells implanted in mice in the control group, CD8^+^ T‐cell treatment group and cocultured CD8^+^ T‐cell treatment group on Day 5 and day 10 and statistical analysis of the above BLI results (*n* = 3). (**I**) Tumour volumes in the control group, CD8^+^ T‐cell treatment group, and cocultured CD8^+^ T‐cell treatment group were assessed on Days 5, 10 and 20 (*n* = 3). Tumour volumes were calculated according to the formula as mentioned. (J) Cumulative Kaplan–Meier survival curves for mice in the control group, CD8^+^ T‐cell treatment group and cocultured CD8^+^ T‐cell treatment group (*n* = 3, log‐rank test). Values are expressed as the means ± SD. Student's *t*‐test was used for two group comparisons (C), and one‐way ANOVA was used to compare three groups (F, G, H, I). The Kaplan–Meier method was used to estimate the survival functions, and the log‐rank test was used for group comparisons (J). *p < .05; BLI: bioluminescent intensity; NS, not significant

To achieve PCa‐specific recognition, we cocultured DC‐CD8^+^ T cells with C4‐2 cells and produced CD8^+^ T cells that specifically recognised C4‐2 cells through adaptive immune memory. During the culturing process, we observed that T cells accounted for 56.1% of PBMCs, and CD8^+^ and CD4^+^ T cells represented 30.3% and 59.3% of T cells, respectively (Figure 7A). Upon stimulation with anti‐CD3 antibody and other cytokines, the proportion of T cells in the PBMCs increased to 96.8%, and the proportion of CD8^+^ T cells increased to 72.8% of T cells before negative selection (Figure 7B). The CD3^+^ T‐cell percentages in PBMCs and CD8^+^ T‐cell percentages in the selected T cells of PBMCs increased significantly after culturing with anti‐CD3 antibody and cytokines (Figure 7C,D). These isolations and cultures yielded cells that were consistently > 90% positive for CD8^+^ T cells (Figure 7E). Next, the CCK results showed that only CD8^+^ T cells cocultured with DCs and C4‐2 cells, rather than those without cocultures, or those cocultured with DCs displayed rapidly specific and powerful cytotoxicity against C4‐2 cells compared with PC3 cells (Figure 7F,G). We then compared the difference in in vivo antitumour effects between CD8^+^ T cells cocultured with DCs and C4‐2 and CD8^+^ T cells alone. The BLI from the tumours in each group showed no significant difference on Day 5, and treatment with 1 × 10^7^ cocultured CD8^+^ T or CD8^+^ T‐cell–cell infusions was administered to each group on Day 5. We re‐examined the BLI on Day 10 and observed that the cocultured CD8^+^ T‐cell group showed dramatically lower BLI and volume than the CD8^+^ T‐cell group and the control group (Figure 7H,I). Moreover, mice treated with cocultured CD8^+^ T cells showed a significant improvement in survival (*p* = .02) (Figure 7J). Taken together, we successfully produced CD8^+^ T cells that can specifically recognize PCa cells, which were used for further research.

### 3.7 Atezolizumab or nivolumab enhanced the in vivo antitumour effect of CAR NK‐92 cells in the presence of cocultured CD8^+^ T cells

The BLIs of the tumours in each group did not show significant differences on Day 7 after C4‐2^GFP^ cells were subcutaneously implanted. Cocultured CD8^+^ T cells were administered on Day 17 on the basis of CAR NK‐92 combined without or with atezolizumab/nivolumab therapy. As predicted, on Day 21, the BLI of tumours treated with cocultured CD8^+^ T cells combined with CAR NK‐92 cells and atezolizumab or nivolumab was evidently lower than that of the tumour treated with a combination of CAR NK‐92 cells and cocultured CD8^+^ T cells without ICI (Figure 6A,C). Our data did not show a significant difference in BLI between the atezolizumab and nivolumab groups when combined with CAR NK‐92 cells and cocultured CD8^+^ T cells (Figure 6A,C), which can be explained by the powerful tumouricidal function of the ICI‐treated CD8^+^ T cells, which were exempt from exhaustion. Consistent with the therapeutic effect reflected by BLI, the volumes and masses of tumours treated with cocultured CD8^+^ T cells combined with CAR NK‐92 and atezolizumab or nivolumab decreased significantly compared to those of tumours treated with combined therapy without ICI (Figure 6G–I). The mice treated with CAR NK‐92 plus atezolizumab demonstrated obviously longer survival times than their counterparts treated with CAR NK‐92 cells or CAR NK‐92 cells plus nivolumab (*p* = .03 for both groups). Moreover, atezolizumab significantly extended the survival time of mice treated with CAR NK‐92 cells and cocultured CD8^+^ T cells (*p* = .04); however, there was no significant difference in survival between the CAR NK92+atezolizumab+cocultured CD8^+^ T and CAR NK92+nivolumab+cocultured CD8^+^ T groups (*p* = .31; Figure 6J).

### 3.8 Atezolizumab enhanced the in vitro cytotoxicity of CAR NK‐92 cells against primary PCa cells obtained from a patient with CRPC

**FIGURE 8:**
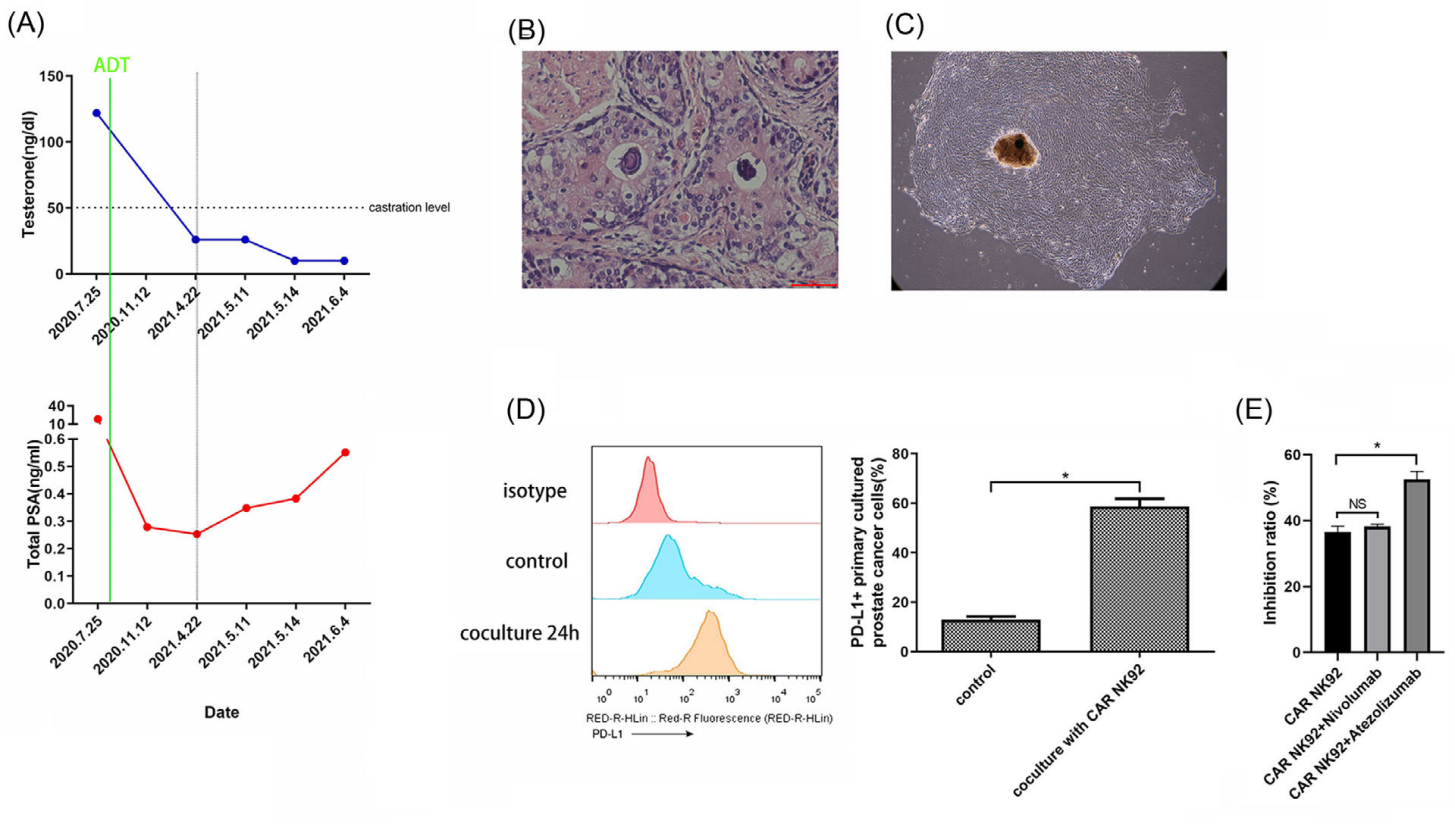
Atezolizumab enhanced the cytotoxicity of CAR NK‐92 against castration‐resistant prostate cancer (CRPC) cells from one patient in vitro. (A) Serum testosterone and PSA level fluctuations after androgen deprivation therapy (ADT) in one CRPC patient. The PSA level began to increase consecutively over the nadir from May 2021 under the castration level of testosterone after ADT. (B) Representative HE‐stained tumour sections from the patient after radical prostatectomy (×200). (C) Light micrographs of primary prostate cancer (PCa) cells on day 14 (×100). (D) Representative flow cytometry plots and summary data (*n* = 3) showing the expression of PD‐L1 on primary PCa cells cocultured with CAR NK‐92 cells for 24 h. (E), CCK‐8 assay result (*n* = 3) showing that atezolizumab (20 μg/ml) but not nivolumab (20 μg/ml) significantly enhanced the inhibition ratio of CAR NK‐92 cells. Data are expressed as the means ± SD. Student's *t*‐test was used for two‐group comparisons (D), and ANOVA was used to compare three groups (E). *p < .05; NS, not significant

The patient's medical history showed that the PSA level had begun to increase consecutively since May 2021 under a castration level of testosterone. The patient was diagnosed with mCRPC according to the American Urological Association guidelines (Figure 8A). Postoperative histopathology revealed a Gleason Grade 9 (4 + 5) adenocarcinoma (Figure 8B). We successfully cultured and acquired primary PCa cells from patient CRPC specimens (Figure 8C). Then, we observed that the expression of PD‐L1 on primary PCa cells was markedly upregulated after coincubation with CAR NK‐92 cells for 24 h (Figure 8D). Moreover, the CCK‐8 results indicated that atezolizumab increased the inhibition ratio of CAR NK‐92 cells when cocultured with primary PCa cells (Figure 8E). Collectively, atezolizumab enhanced the in vitro cytotoxicity of CAR NK‐92 cells against primary PCa cells acquired from a patient with CRPC, which was consistent with the results obtained from the C4‐2 cell line cocultured with CAR NK‐92 cells.

## 4 DISCUSSION

We demonstrated that atezolizumab enhanced both the in vitro and in vivo antitumour effects of our newly designed anti‐PSMA CAR NK‐92 cells against CRPC via two completely different mechanisms: blockade of the PD‐L1/PD‐1 axis and direct activation of PD‐L1‐expressing CAR NK‐92. We also revealed the different mechanisms of PD‐L1 upregulation in cocultured CAR NK‐92 and CRPC cells. To date, we are the first to explore the antitumour efficacy of combined CAR NK‐92 cells and anti‐PD‐L1 mAb therapy against CRPC and the first to unravel a unique PD‐1‐independent mechanism by which CAR NK‐92 cells are activated by anti‐PD‐L1 mAb. Our study has clinical implications regarding the application of anti‐PD‐L1 mAb in patients with CRPC currently undergoing CAR NK‐92 cell therapy to synergistically combat cancer cells. Moreover, our study provided novel clues to the possible mechanisms behind the direct activation of immune cells by anti‐PD‐L1 mAb and explained why some patients without expression of PD‐L1 on their tumours still responded to anti‐PD‐L1 mAb treatment.

IFN‐γ is secreted by NK and T cells after activation, and it can induce tumour cells to express PD‐L1.^9^, ^27^ PD‐L1 can inactivate T cells when bound to PD‐1 on activated T cells.^28^ Moreover, PD‐L1 on tumour cells can be upregulated as part of adaptive resistance upon encountering activated T and NK cells.^29^, ^30^, ^31^, ^32^, ^33^ Based on these premises, we hypothesised that IFN‐γ secreted by CAR NK‐92 cells might upregulate PD‐L1 expression on C4‐2 cells. Then, we observed that CAR NK‐92 cells secreted IFN‐γ time‐dependently after encountering C4‐2 cells, and the expression of PD‐L1 on C4‐2 cells was upregulated significantly in a time‐dependent manner after coculture. In addition, we stimulated target cells with IFN‐γ and showed that the expression of PD‐L1 on target cells increased in an IFN‐γ concentration‐dependent manner. Moreover, when IFN‐γ was neutralised completely with a blocking antibody in coculture medium, C4‐2 cell expression of PD‐L1 was no longer elevated, which further proved our hypothesis. Collectively, our findings provide solid proof that the PD‐L1 upregulation in C4‐2 cells was solely dependent on IFN‐γ secreted by cocultured CAR NK‐92 cells. IFN‐receptor binding at the cell surface triggers the activation of the JAK‐STAT pathway, which modulates the upregulation of PD‐L1.^34^, ^35^ Our KEGG analysis based on RNA sequencing of cocultured C4‐2 cells and C4‐2 cells alone indicated that the JAK‐STAT signal transduction pathway was activated, which was then validated by our western blotting results showing the activated JAK1/2 and STAT1 proteins in cocultured C4‐2 cells.

As we have already observed upregulation of PD‐L1 expression in C4‐2 cells after CAR NK‐92 cell treatment and elucidated its mechanisms, we postulated that anti‐PD‐L1/PD‐1 mAb would reinvigorate exhausted T cells expressing PD‐1 on their surface bound by PD‐L1 from PCa cells. CD8^+^ T cells cocultured with DCs and C4‐2 cells gained specific recognition ability to partly simulate the TME in vivo. Consistent with our expectations, both atezolizumab and nivolumab restored the cytolytic effect of cocultured CD8^+^ T cells in a mouse model of CRPC.

In addition to C4‐2 cell upregulation of PD‐L1, we noticed that PD‐L1 on CAR NK‐92 cells time‐dependently increased when cocultured with C4‐2 cells. Therefore, we posited that there were two possibilities for this phenomenon: either PD‐L1 upregulation in CAR NK‐92 cells was dependent on direct cell‐to‐cell contact or dependent on cytokines in the culture medium other than IFN‐γ. We observed that the conditioned media did not induce PD‐L1 upregulation, and C4‐2 cells incubated at the bottom of the dish did not cause PD‐L1 upregulation in CAR NK‐92 cells incubated in trans‐wells. Collectively, direct physical contact between CAR NK‐92 and C4‐2 cells is a prerequisite for CAR NK‐92 cell upregulation of PD‐L1. Some evidence suggests that the tumour and immune cells can regulate PD‐L1 expressions in different ways. Noguchi et al. showed that IFN‐γ‐blocking antibodies therapy substantially eliminated PD‐L1 expression in cancer cells but partially decreased that in macrophages in a sarcoma mouse model.^36^ In line with our findings, one recent study conducted by Dong et al. confirmed that the expression of PD‐L1 on peripheral NK cells was induced when NK cells were cocultured with K562, a myeloid leukaemia cell line, and this induction required direct cell‐to‐cell contact.^14^

To further explore the mechanisms of C4‐2‐induced PD‐L1 upregulation in CAR NK‐92 cells, we performed RNA sequencing and KEGG analysis, which revealed that the PI3K/AKT/mTOR pathway was remarkably enriched, while the IFN‐γ‐mediated JAK‐STAT pathway was downregulated in activated CAR NK‐92 cells, compared to their quiescent counterparts. Furthermore, we confirmed the central role of the PI3K/AKT/mTOR pathway in upregulating PD‐L1 using western blotting. After comprehensive analysis of all signalling pathways regulating PD‐L1 expression in the TME^22^, ^27^ and all NK‐cell activation pathways,^23^ we inferred that PD‐L1 upregulation in activated NK‐92 cells was mediated through the NKG2D/PI3K/AKT/mTOR pathway when the activating receptor NKG2D on cell surfaces bound to MICA/B expressed abundantly in C4‐2 cells. We found that treatment with an NKG2D blocker significantly reduced the expression of PD‐L1 and PI3K/AKT/mTOR‐related phosphorylated proteins in NK‐92 cells. However, the CAR NK‐92 cell expression of these signalling proteins could not be inhibited by an NKG2D blocker because the activation of CAR NK‐92 cells mainly depends on recognition of PSMA and its subsequent intracellular signal transduction mediated by our designed CD244‐NKG2D intracellular segment, rather than the recognition of MICA/B by membrane NKG2D. Next, we demonstrated that PI3K and AKT inhibitors were capable of blocking PD‐L1 expression in effector cells. The current study first revealed that CAR NK‐92 cells, upon encountering and being activated by C4‐2 cells, not only produce amounts of IFN‐γ and release lytic granules but also modulate the PD‐L1 expression on their surfaces via the NKG2D/PI3K/AKT/mTOR signalling pathway. Previously, some reports have focused on the function of the PI3K pathway in regulating PD‐L1 expression. Dong et al. reported that the PI3K/AKT/NF‐κB pathway mediated the PD‐L1 upregulation in NK cells after being activated by NK‐sensitive target cells, and subunit p65 of NF‐κB, the final effector molecule, regulated the expression of PD‐L1.^14^ The findings of the PI3K pathway‐mediated regulation of PD‐L1 expression were consistent with our observation. However, it did not mention the upstream signalling molecule of the PI3K pathway, and the effector molecule was NF‐κB, which was different from our findings. Karakhanova et al. reported that monocyte‐derived plasmacytoid DC can utilize the PI3K pathway to upregulate PD‐L1 in response to cytokines.^37^ Muthumani et al. found that the PI3K signalling pathway plays crucial roles in upregulating PD‐L1 expression in macrophages and DCs following human immunodeficiency virus (HIV) infection and that inhibition of PI3K signal transducer enzymes obviously reduced the induction of PD‐L1.^38^ In addition to antigen‐presenting cells (APC), PI3K pathway activation has also been reported to upregulate the expression of PD‐L1 in several types of cancers.^39^, ^40^, ^41^, ^42^, ^43^ In most of these cancers, activation of the PI3K pathway was caused by PTEN loss or silencing, which was different from our finding that the PI3K pathway was activated via CAR NK‐92 cell activation.

However, the significance and role of PD‐L1 expressed by immune cells have not been clarified. Diskin et al. reported that PD‐L1 on T cell supresses effector T cells and PD‐1+ macrophages.^10^ Hartley et al. proved that PD‐L1 delivered a constitutively negative signal to macrophages; PD‐L1 antibody enhanced macrophage‐mediated antitumour activity.^11^ Mayoux et al. reported that anti‐PD‐L1 Ab could trigger DCs to relieve CD80 by blocking PD‐L1, which is abundantly expressed on DCs, allowing increased CD80/CD28 interaction to prime T cells.^12^ Iraolagoitia et al. reported that PD‐L1 on NK cells suppresses the activations of CD8^+^ T and DC cells by binding to PD‐1 expressed on these cells.^13^ Dong et al. showed that a PD‐L1 antibody directly enhanced NK‐mediated antitumour activity.^14^ Based on these premises, we assumed that there might be two key points in PD‐L1 upregulation on CAR NK‐92 cells after encountering C4‐2 cells, which requires further validation. On the one hand, PD‐L1 is a marker of CAR NK 92 cell activation; on the other hand, PD‐L1 may promote self‐tolerance and prevent overactivation of the immune system by suppressing neighbouring effector T cells and macrophages in the TME.

At present, there are three PD‐L1 mAbs and three PD‐1 mAbs approved by the FDA for cancer therapy.^44^ We chose the PD‐L1 mAb atezolizumab and PD‐1 mAb nivolumab as interventions in our study. Our results showed that atezolizumab significantly enhanced the in vitro cytotoxicity against CRPC cells and IFN‐γ secretion of CAR NK‐92 cells when compared to CAR NK‐92 cells alone or CAR NK‐92 cells combined with nivolumab. Moreover, the present study demonstrated that atezolizumab significantly augmented the antitumour efficacy of CAR NK‐92 cells in a mouse tumour model. Only one study reported that mice treated with atezolizumab and human peripheral NK cells bore a significantly lower tumour burden than those treated with only NK cells.^14^ Although the NK cells and tumour cells used in our study were completely different and they did not use PD‐1 mAb as the control, the PD‐L1 mAb atezolizumab enhanced the functions of effector cells in both studies. Interestingly, our team observed that nivolumab marginally enhanced the cytotoxicity and antitumour effect of CAR NK‐92 cells both in vitro and in vivo. This phenomenon can be explained as follows: the expression of PD‐1 on a small proportion of these effector cells was upregulated after coculturing with C4‐2 cells, and nivolumab reversed the exhaustion of these cells. In our study, CAR NK‐92 cells did not constitutively express PD‐1, and the mild elevation of the immune checkpoint PD‐1 was caused by interaction with tumour cells. Hasim et al. found that NK‐cell expression of PD‐1 was upregulated after coculturing with two PD‐1‐expressing leukaemia cell lines, and they proved that NK cells acquired PD‐1 by trogocytosis.^45^, ^46^ Our discovery of PD‐1 upregulation in CAR NK‐92 cells requires further exploration.

To explore the effect of atezolizumab combined with CAR NK‐92 cells against CRPC with the existence of CD8^+^ T cells, we adopted the cocultured CD8^+^ T cells to simulate the human TME in our CRPC mouse model. DCs are the most potent APC and can present antigenic epitopes to CD8^+^ T cells after processing the loaded tumour antigens, resulting in the induction of specific antitumour immunity of CD8^+^ T cells.^47^ Some studies have used many different forms of tumour cells (including necrotic, apoptotic and live cells) and tumour tissue lysates as antigens to load DCs to improve the cytotoxicity of CD8^+^ T cells.^48^, ^49^, ^50^, ^51^, ^52^ In our study, we incubated CD8^+^ T cells and DCs with targeted C4‐2 cells and acquired cocultured CD8^+^ T cells that were supposed to have a specific tumour‐killing function, which was validated by comparing BLIs, tumour volumes and survival time in different treatment groups in vivo. In our study, the administration of cocultured CD8^+^ T cells on the basis of CAR NK‐92 cells with atezolizumab or nivolumab treatment significantly improved the therapeutic efficacy against CRPC in vivo when compared to treatment without ICI, which was in accordance with our prediction. However, as demonstrated by the tumour size and survival time, atezolizumab did not show any evident therapeutic advantage over nivolumab with cocultured CD8^+^ T cells. The reason behind this phenomenon could be that the advantage of atezolizumab was overshadowed by the powerful specific killing of cocultured CD8^+^ T cells unleashed by ICI.

In our study, we mainly used the human cell line C4‐2 with high PSMA expression as the target tumour cells. In addition, we acquired PCa specimens and successfully performed primary cell cultures from a patient with CRPC who underwent RARP. The data showed that the expression of PD‐L1 in primary cancer cells increased dramatically after coculture with CAR NK‐92 cells, and atezolizumab enhanced the cytotoxicity of CAR NK‐92 cells against primary PCa cells in vitro. These findings were consistent with the results obtained from the coculture of CAR NK‐92 cells with C4‐2 cells and further supported our discovery from a clinical perspective.

## 5 CONCLUSION

**FIGURE 9:**
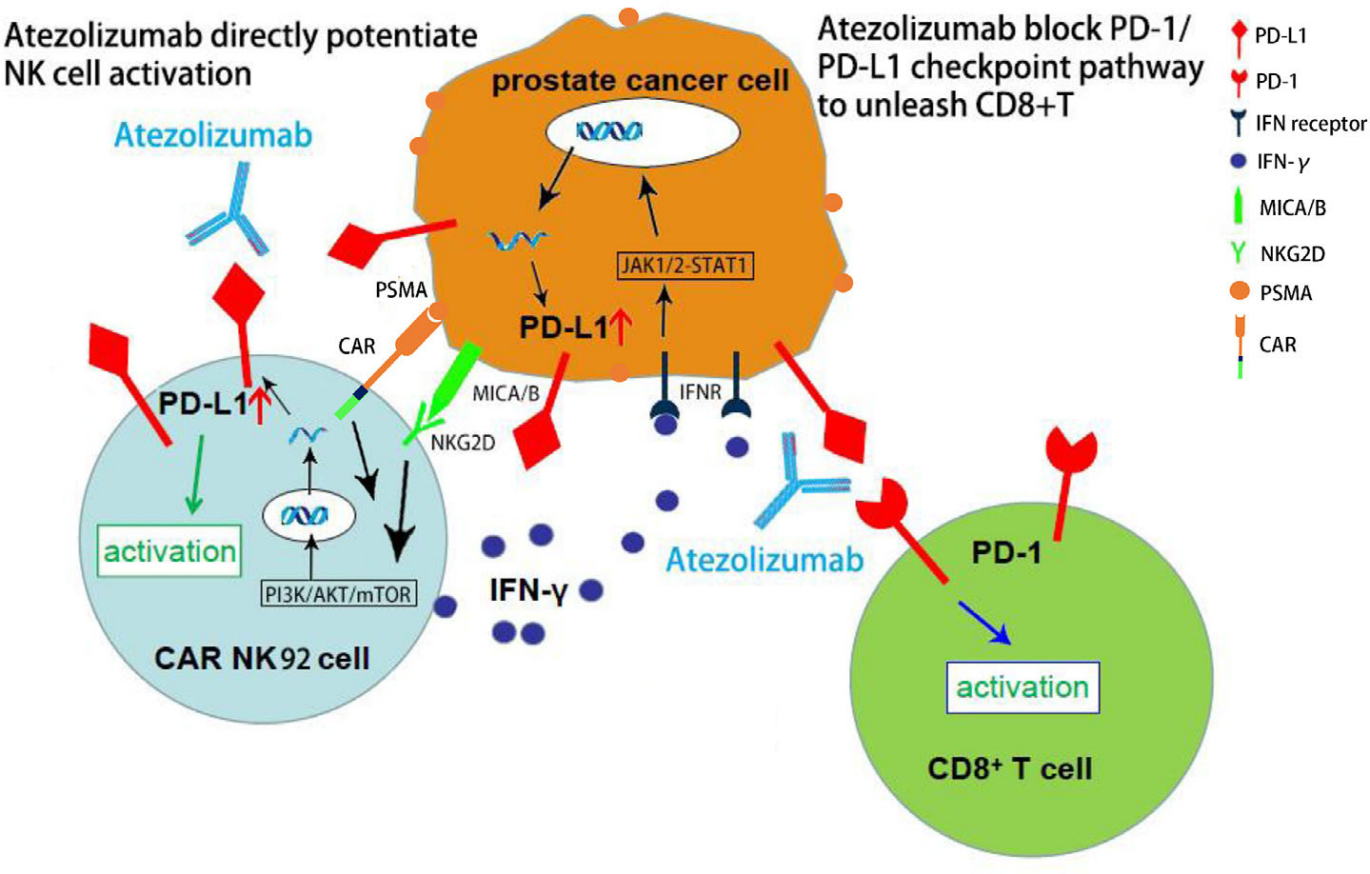
Summary diagram of our results. PSMA‐specific CAR‐modified NK92 cells were activated through the specific recognition of PSMA on PCa cells, and CAR NK‐92 cell expression of PD‐L1 was upregulated via the PI3K/AKT/mTOR signalling pathway, while C4‐2 cell expression of PD‐L1 was upregulated via the IFN‐γ‐mediated JAK1/2‐STAT1 pathway. On the one hand, atezolizumab potentiated CAR NK‐92 cell cytotoxicity by directly acting on PD‐L1 expressed on CAR NK‐92 cells; on the other hand, atezolizumab unleashed CD8^+^ T cells by blocking the canonical PD‐L1/PD‐1 axis; MICA/B, MHC class I chain‐related protein A and B; NKG2D, NK group 2 member D; PSMA, prostate‐specific membrane antigen

In summary (Figure 9), our study demonstrated that PD‐L1 expressions on both C4‐2 and CAR NK‐92 cells were upregulated during coculturing via different mechanisms; in the former, it was mediated by IFN‐γ via the JAK1/2‐STAT1 signalling pathway, while in the latter, it was mainly dependent on direct cell‐to‐cell interaction via the CAR‐mediated NKG2D/PI3K/AKT pathway. We demonstrated that the binding of atezolizumab to PD‐L1, which was upregulated on the surfaces of CAR NK‐92 cells, induced stronger in vitro and in vivo antitumour activity by a novel mechanism independent of the canonical PD‐L1/PD‐1 axis and in the absence of any other immune cells. Moreover, we successfully enhanced the antitumour effect of CAR NK‐92 cells with cocultured CD8^+^ T cells in vivo by blocking the PD‐L1/PD‐1 axis using atezolizumab or nivolumab. Collectively, our experimental data suggest that a combination of anti‐PSMA CAR NK‐92 cells and anti‐PD‐L1 mAb enhanced the antitumour efficacy against CRPC, which has great value in clinical applications for patients with CRPC. Finally, the data from our study provide important clues to the significance of PD‐L1 expression in immune effector cells, including NK, T and B cells and macrophages. The clinical application of CAR NK‐92 cells combined with anti‐PD‐L1 mAb therapy represents a promising immunotherapeutic strategy for the treatment of patients with CRPC.

## CONFLICT OF INTEREST

The authors declare that they have no competing interests.

## Supporting information

Supporting Information

Supporting Information

Supporting Information

Supporting Information

Supporting Information

Supporting Information

## References

[1] Sung H , Ferlay J , Siegel RL , et al. Global cancer statistics 2020: GLOBOCAN estimates of incidence and mortality worldwide for 36 cancers in 185 countries. CA Cancer J Clin. 2021;71(3):209‐249. 33538338 10.3322/caac.21660

[2] Litwin MS , Tan HJ . The diagnosis and treatment of prostate cancer: a review. JAMA. 2017;317(24):2532‐2542. 28655021 10.1001/jama.2017.7248

[3] Katsogiannou M , Ziouziou H , Karaki S , Andrieu C , Henry de Villeneuve M , Rocchi P . The hallmarks of castration‐resistant prostate cancers. Cancer Treat Rev. 2015;41(7):588‐597. 25981454 10.1016/j.ctrv.2015.05.003

[4] Schepisi G , Cursano MC , Casadei C , et al. CAR‐T‐cell therapy: a potential new strategy against prostate cancer. J Immunother Cancer. 2019;7(1):258. 31619289 10.1186/s40425-019-0741-7 PMC6794851

[5] Bilusic M , Madan RA , Gulley JL . Immunotherapy of prostate cancer: facts and hopes. Clin Cancer Res. 2017;23(22):6764‐6770. 28663235 10.1158/1078-0432.CCR-17-0019 PMC5690854

[6] Alzubi J , Dettmer‐Monaco V , Kuehle J , et al. PSMA‐directed CAR T cells combined with low‐dose docetaxel treatment induce tumor regression in a prostate cancer xenograft model. Mol Ther Oncolytics. 2020;18:226‐235. 32728611 10.1016/j.omto.2020.06.014 PMC7372156

[7] Park JH , Rivière I , Gonen M , et al. Long‐term follow‐up of CD19 CAR therapy in acute lymphoblastic leukemia. N Engl J Med. 2018;378(5):449‐459. 29385376 10.1056/NEJMoa1709919 PMC6637939

[8] Xie G , Dong H , Liang Y , Ham JD , Rizwan R , Chen J . CAR‐NK cells: a promising cellular immunotherapy for cancer. EBioMedicine. 2020;59:102975. 32853984 10.1016/j.ebiom.2020.102975 PMC7452675

[9] Alspach E , Lussier DM , Schreiber RD . Interferon γ and its important roles in promoting and inhibiting spontaneous and therapeutic cancer immunity. Cold Spring Harb Perspect Biol. 2019;11(3):a028480. 29661791 10.1101/cshperspect.a028480 PMC6396335

[10] Diskin B , Adam S , Cassini MF , et al. PD‐L1 engagement on T cells promotes self‐tolerance and suppression of neighboring macrophages and effector T cells in cancer. Nat Immunol. 2020;21(4):442‐454. 32152508 10.1038/s41590-020-0620-x

[11] Hartley GP , Chow L , Ammons DT , Wheat WH , Dow SW . Programmed cell death ligand 1 (PD‐L1) signaling regulates macrophage proliferation and activation. Cancer Immunol Res. 2018;6(10):1260‐1273. 30012633 10.1158/2326-6066.CIR-17-0537

[12] Mayoux M , Roller A , Pulko V , et al. Dendritic cells dictate responses to PD‐L1 blockade cancer immunotherapy. Sci Transl Med. 2020;12(534):eaav7431. 32161104 10.1126/scitranslmed.aav7431

[13] Iraolagoitia XL , Spallanzani RG , Torres NI , et al. NK cells restrain spontaneous antitumor CD8^+^ T‐cell priming through PD‐1/PD‐L1 Interactions with dendritic cells. J Immunol. 2016;197(3):953‐961. 27342842 10.4049/jimmunol.1502291

[14] Dong W , Wu X , Ma S , et al. The mechanism of anti‐PD‐L1 antibody efficacy against PD‐L1‐negative tumors identifies NK cells expressing PD‐L1 as a cytolytic effector. Cancer Discov. 2019;9(10):1422‐1437. 31340937 10.1158/2159-8290.CD-18-1259 PMC7253691

[15] Zuccolotto G , Fracasso G , Merlo A , et al. PSMA‐specific CAR‐engineered T cells eradicate disseminated prostate cancer in preclinical models. PLoS One. 2014;9(10):e109427. 25279468 10.1371/journal.pone.0109427 PMC4184866

[16] Montagner IM , Penna A , Fracasso G , et al. Anti‐PSMA CAR‐engineered NK‐92 cells: an off‐the‐shelf cell therapy for prostate cancer. Cells. 2020;9(6):1382. 10.3390/cells9061382 PMC7349573 32498368

[17] Zhang M , Wang P , Luo R , et al. Biomimetic human disease model of SARS‐CoV‐2 induced lung injury and immune responses on organ chip system. Adv Sci (Weinh). 2020;8(3):2002928. 10.1002/advs.202002928 PMC7646023 33173719

[18] Guo Y , Luo R , Wang Y , et al. SARS‐CoV‐2 induced intestinal responses with a biomimetic human gut‐on‐chip. Sci Bull (Beijing). 2021;66(8):783‐793. 33282445 10.1016/j.scib.2020.11.015 PMC7704334

[19] Song C , Shi D , Chang K , et al. Sodium fluoride activates the extrinsic apoptosis via regulating NOX4/ROS‐mediated p53/DR5 signaling pathway in lung cells both in vitro and in vivo. Free Radic Biol Med. 2021;169:137‐148. 33857626 10.1016/j.freeradbiomed.2021.04.007

[20] Xiang T , Li J , Bao S , et al. Digital RNA‐seq transcriptome plus tissue anatomy analyses reveal the developmental mechanism of the calabash‐shaped root in *Tetrastigma hemsleyanum* . Tree Physiol. 2021;41(9):1729‐1748. 33601408 10.1093/treephys/tpab024

[21] Wang F , Dong X , Wang J , Yang F , Liu D , Ma J , et al. Allogeneic expanded human peripheral NK cells control prostate cancer growth in a preclinical mouse model of castration‐resistant prostate cancer. J Immunol Res. 2022;2022:1786395. 10.1155/2022/1786395 35450395 PMC9017519

[22] Yi M , Niu M , Xu L , Luo S , Wu K . Regulation of PD‐L1 expression in the tumor microenvironment. J Hematol Oncol. 2021;14(1):10. 33413496 10.1186/s13045-020-01027-5 PMC7792099

[23] Vivier E , Nunès JA , Vély F . Natural killer cell signaling pathways. Science. 2004;306(5701):1517‐1519. 15567854 10.1126/science.1103478

[24] Santini FC , Rudin CM . Atezolizumab for the treatment of non‐small‐cell lung cancer. Expert Rev Clin Pharmacol. 2017;10(9):935‐945. 28714780 10.1080/17512433.2017.1356717 PMC6089509

[25] Weinstock C , Khozin S , Suzman D , et al. U.S. food and drug administration approval summary: atezolizumab for metastatic non–small cell lung cancer. Clin Cancer Res. 2017;23(16):4534‐4539. 28611199 10.1158/1078-0432.CCR-17-0540

[26] Ferris RL , Blumenschein G Jr , Fayette J , et al. Nivolumab for recurrent squamous‐cell carcinoma of the head and neck. N Engl J Med. 2016;375(19):1856‐1867. 27718784 10.1056/NEJMoa1602252 PMC5564292

[27] Sun C , Mezzadra R , Schumacher TN . Regulation and function of the PD‐L1 checkpoint. Immunity. 2018;48(3):434‐452. 29562194 10.1016/j.immuni.2018.03.014 PMC7116507

[28] Fife BT , Pauken KE , Eagar TN , et al. Interactions between PD‐1 and PD‐L1 promote tolerance by blocking the TCR‐induced stop signal. Nat Immunol. 2009;10(11):1185‐1192. 19783989 10.1038/ni.1790 PMC2778301

[29] Pardoll DM . The blockade of immune checkpoints in cancer immunotherapy. Nat Rev Cancer. 2012;12(4):252‐264. 22437870 10.1038/nrc3239 PMC4856023

[30] Kamphorst AO , Wieland A , Nasti T , et al. Rescue of exhausted CD8 T cells by PD‐1‐targeted therapies is CD28‐dependent. Science. 2017;355(6332):1423‐1427. 28280249 10.1126/science.aaf0683 PMC5595217

[31] Mandai M , Hamanishi J , Abiko K , Matsumura N , Baba T , Konishi I . Dual faces of IFN gamma in cancer progression: a role of PD‐L1 induction in the determination of pro‐ and antitumor immunity. Clin Cancer Res. 2016;22(10):2329‐2334. 27016309 10.1158/1078-0432.CCR-16-0224

[32] Spranger S , Spaapen RM , Zha Y , et al. Up‐regulation of PD‐L1, IDO, and T(regs) in the melanoma tumor microenvironment is driven by CD8 (+) T cells. Sci Transl Med. 2013;5(200):200ra116. 10.1126/scitranslmed.3006504 PMC4136707 23986400

[33] Oyer JL , Gitto SB , Altomare DA , Copik AJ . PD‐L1 blockade enhances anti‐tumor efficacy of NK cells. Oncoimmunology. 2018;7(11):e1509819. 30377572 10.1080/2162402X.2018.1509819 PMC6205063

[34] Platanias LC . Mechanisms of type‐I‐ and type‐II‐interferon‐mediated signalling. Nat Rev Immunol. 2005;5(5):375‐386. 15864272 10.1038/nri1604

[35] Lee SJ , Jang BC , Lee SW , et al. Interferon regulatory factor‐1 is prerequisite to the constitutive expression and IFN‐gamma‐induced upregulation of B7‐H1 (CD274). FEBS Lett. 2006;580(3):755‐762. 16413538 10.1016/j.febslet.2005.12.093

[36] Noguchi T , Ward JP , Gubin MM , et al. Temporally distinct PD‐L1 expression by tumor and host cells contributes to immune escape. Cancer Immunol Res. 2017;5(2):106‐117. 28073774 10.1158/2326-6066.CIR-16-0391 PMC5510474

[37] Karakhanova S , Meisel S , Ring S , Mahnke K , Enk AH . ERK/p38 MAP‐kinases and PI3K are involved in the differential regulation of B7‐H1 expression in DC subsets. Eur J Immunol. 2010;40(1):254‐266. 19830728 10.1002/eji.200939289

[38] Muthumani K , Shedlock DJ , Choo DK , et al. HIV‐mediated phosphatidylinositol 3‐kinase/serine‐threonine kinase activation in APCs leads to programmed death‐1 ligand upregulation and suppression of HIV‐specific CD8 T cells. J Immunol. 2011;187(6):2932‐2943. 21856939 10.4049/jimmunol.1100594 PMC3197696

[39] Parsa AT , Waldron JS , Panner A , et al. Loss of tumor suppressor PTEN function increases B7‐H1 expression and immunoresistance in glioma. Nat Med. 2007;13(1):84‐88. 17159987 10.1038/nm1517

[40] Song M , Chen D , Lu B , et al. PTEN loss increases PD‐L1 protein expression and affects the correlation between PD‐L1 expression and clinical parameters in colorectal cancer. PLoS ONE. 2013;8(6):e65821. 23785454 10.1371/journal.pone.0065821 PMC3681867

[41] Xu C , Fillmore CM , Koyama S , et al. Loss of Lkb1 and Pten leads to lung squamous cell carcinoma with elevated PDL1 expression. Cancer Cell. 2014;25(5):590‐604. 24794706 10.1016/j.ccr.2014.03.033 PMC4112370

[42] Lastwika KJ , Wilson W 3rd , Li QK , et al. Control of PD‐L1 expression by oncogenic activation of the AKT‐mTOR pathway in non‐small‐cell lung cancer. Cancer Res. 2016;76(2):227‐238. 26637667 10.1158/0008-5472.CAN-14-3362

[43] Mittendorf EA , Philips AV , Meric‐Bernstam F , et al. PD‐L1 expression in triple‐negative breast cancer. Cancer Immunol Res. 2014;2(4):361‐370. 24764583 10.1158/2326-6066.CIR-13-0127 PMC4000553

[44] Vaddepally RK , Kharel P , Pandey R , Garje R , Chandra AB . Review of indications of FDA‐approved immune checkpoint inhibitors per NCCN guidelines with the level of evidence. Cancers (Basel). 2020;12(3):738. 10.3390/cancers12030738 PMC7140028 32245016

[45] Newman J , Horowitz A . NK cells seize PD1 from leukaemia cells. Nat Rev Immunol. 2021;21(6):345. 33981086 10.1038/s41577-021-00562-7 PMC8293839

[46] MacMillan O , Alonso FG , Burke KP , et al. When killers become thieves: Trogocytosed PD‐1 inhibits NK cells in cancer. Sci Adv. 2022;8(15):eabj3286. 35417234 10.1126/sciadv.abj3286 PMC9007500

[47] Zhou Y , McEarchern JA , Howard E , Pestano G , Salgaller ML , Bosch ML . Dendritic cells efficiently acquire and present antigen derived from lung cancer cells and induce antigen‐specific T‐cell responses. Cancer Immunol Immunother. 2003;52(7):413‐422. 12835918 10.1007/s00262-003-0382-y PMC11034190

[48] Albert ML , Sauter B , Bhardwaj N . Dendritic cells acquire antigen from apoptotic cells and induce class I‐restricted CTLs. Nature. 1998;392(6671):86‐89. 9510252 10.1038/32183

[49] Celluzzi CM . Physical interaction between dendritic cells and tumor cells results in an immunogen that induces protective and therapeutic tumor rejection. J Immunol. 1998;160(7):3081‐3085. 9531260

[50] Kurokawa T , Oelke M , Mackensen A . Induction and clonal expansion of tumor‐specific cytotoxic T lymphocytes from renal cell carcinoma patients after stimulation with autologous dendritic cells loaded with tumor cells. Int J Cancer. 2001;91(6):749‐756. 11275975 10.1002/1097-0215(200002)9999:9999<::aid-ijc1141>3.0.co;2-x

[51] Lambert LA , Gibson GR , Maloney M . Equipotent generation of protective antitumor immunity by various methods of dendritic cell loading with whole cell tumor antigens. J Immunother. 2001;24(3):232‐236. 11394500

[52] Sauter B , Albert ML , Francisco L , Larsson M , Somersan S , Bhardwaj N . Consequences of cell death: exposure to necrotic tumor cells, but not primary tissue cells or apoptotic cells, induces the maturation of immunostimulatory dendritic cells. J Exp Med. 2000;191(3):423‐434. 10662788 10.1084/jem.191.3.423 PMC2195816

