# Supplementary figures and images for: Combined treatment with anti‐PSMA CAR NK‐92 cell and anti‐PD‐L1 monoclonal antibody enhances the antitumour efficacy against castration‐resistant prostate cancer
Source: Clin Transl Med. 2022 Jun 13;12(6):e901. doi: 10.1002/ctm2.901 (PMC9191826; doi:10.1002/ctm2.901)

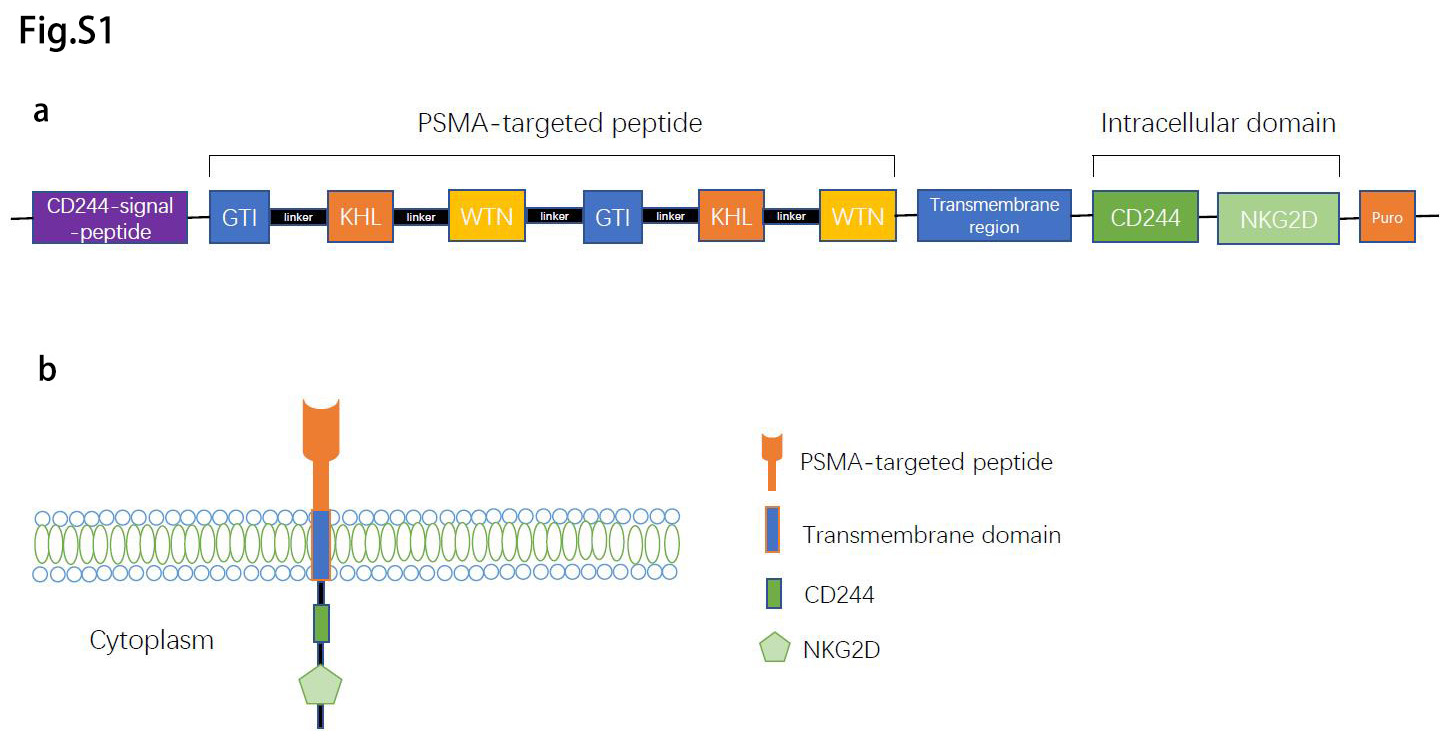

Supplement: Supplementary file 1 — Supporting Information [file CTM2-12-e901-s003.jpg]

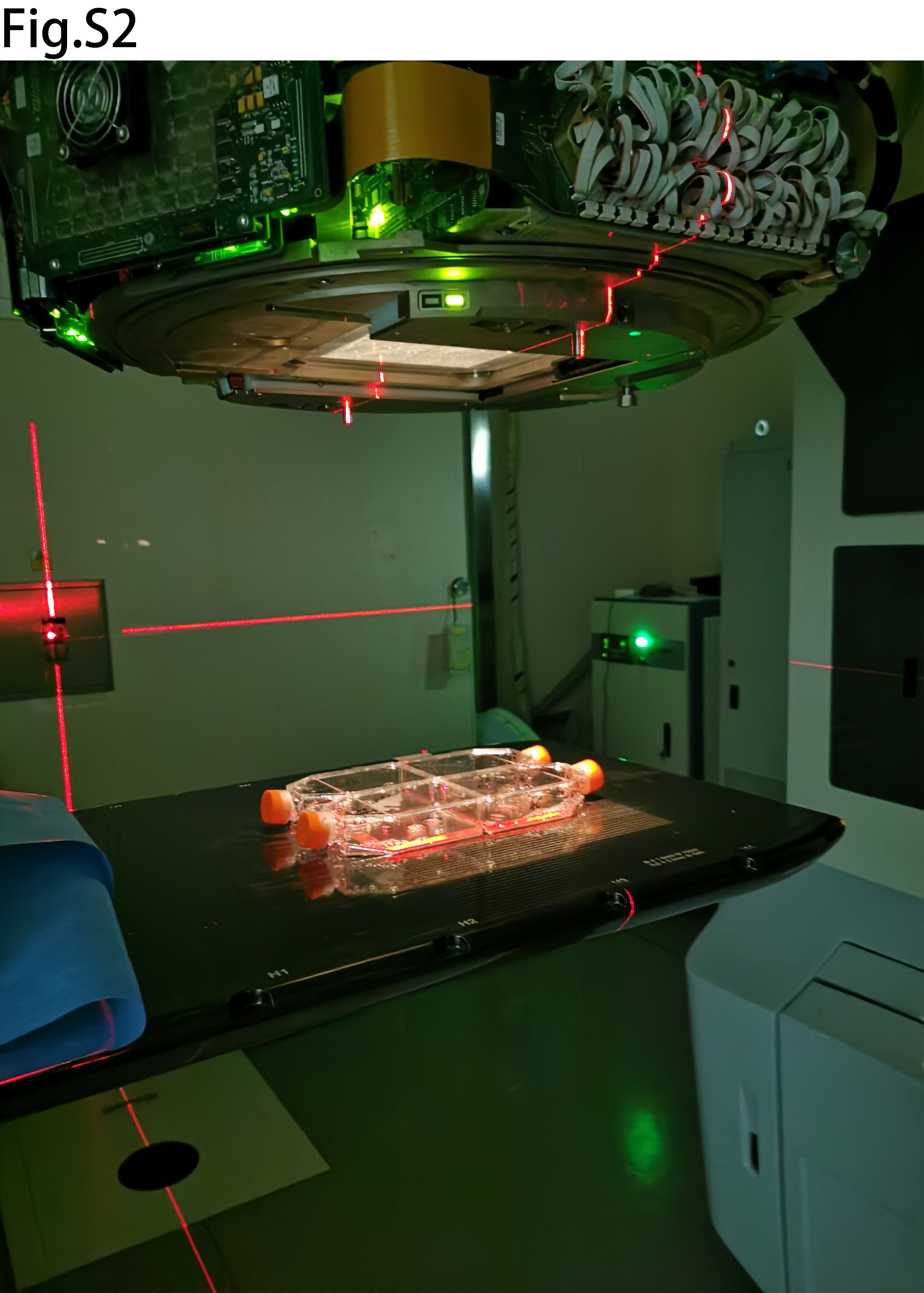

Supplement: Supplementary file 2 — Supporting Information [file CTM2-12-e901-s004.jpg]

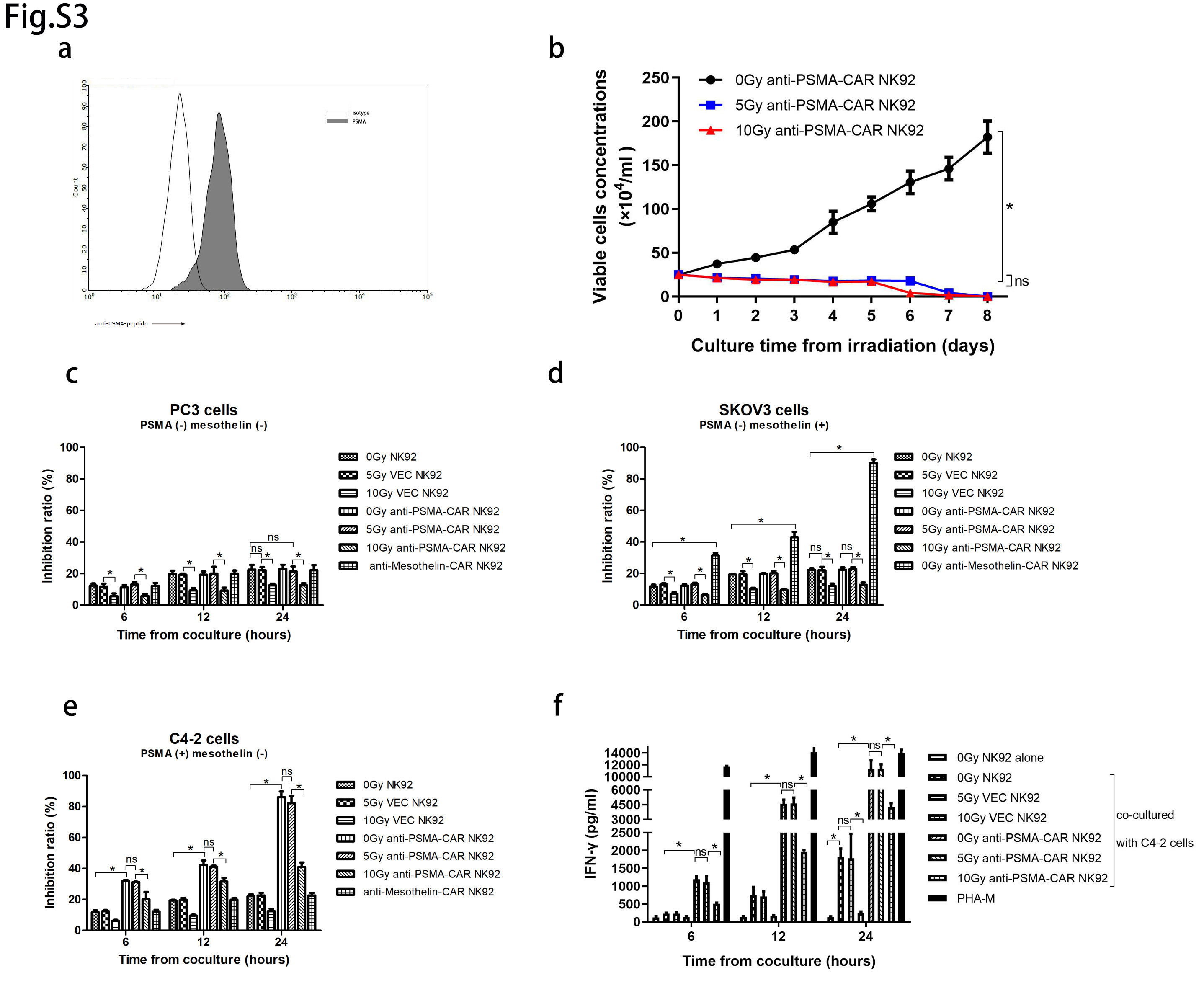

Supplement: Supplementary file 3 — Supporting Information [file CTM2-12-e901-s001.jpg]

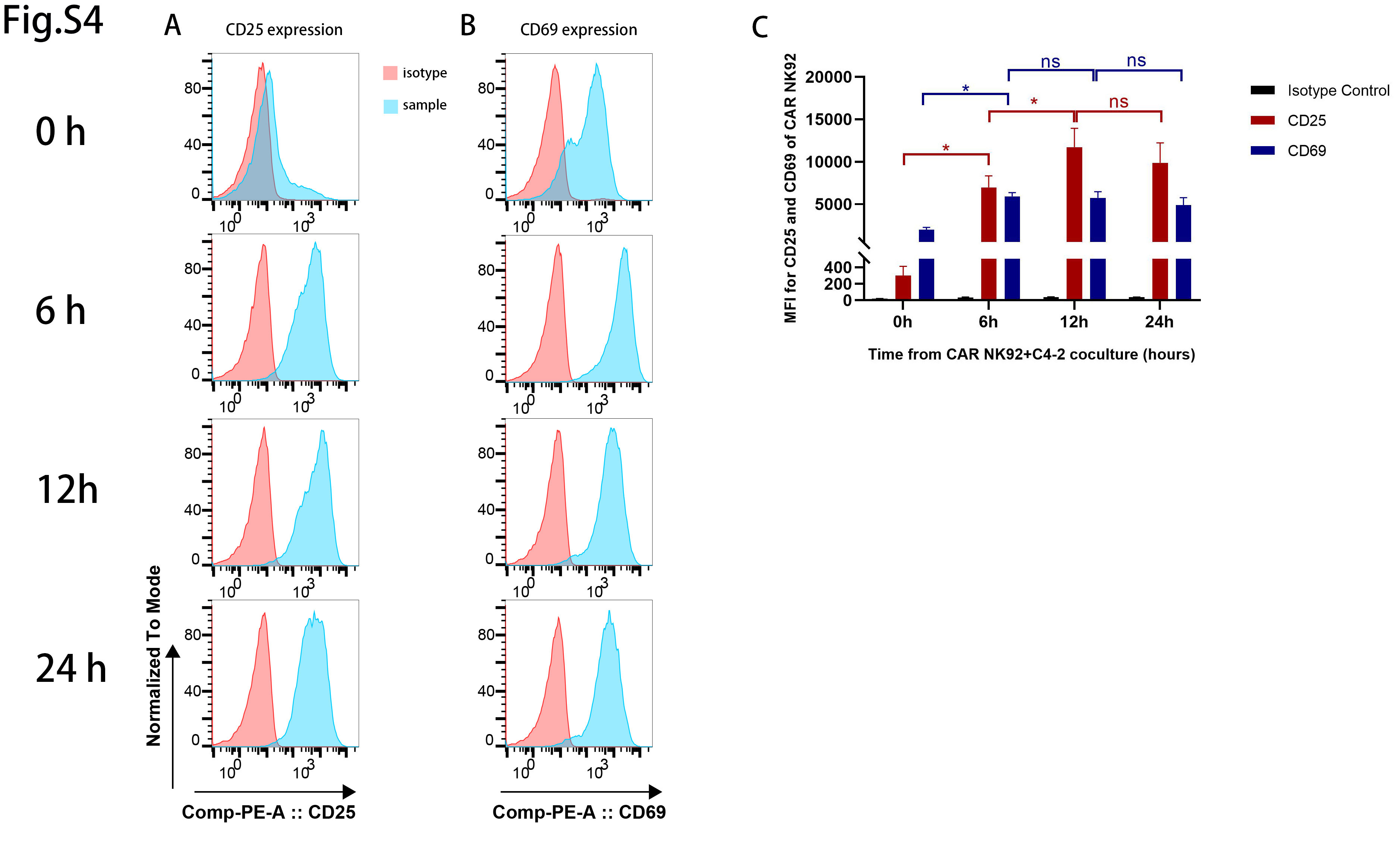

Supplement: Supplementary file 4 — Supporting Information [file CTM2-12-e901-s002.jpg]

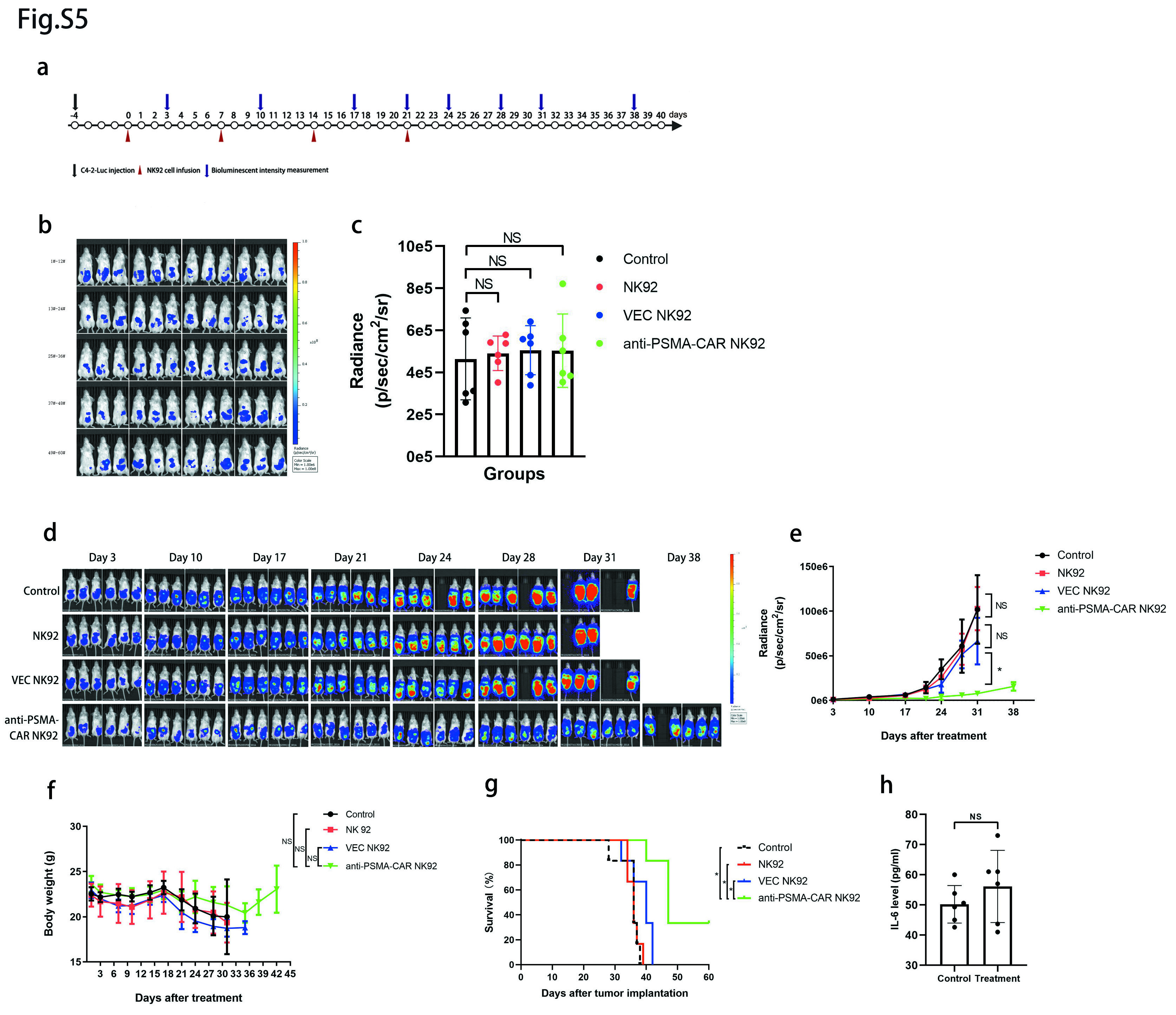

Supplement: Supplementary file 5 — Supporting Information [file CTM2-12-e901-s006.jpg]
