# Supplementary material for: Combined treatment with anti‐PSMA CAR NK‐92 cell and anti‐PD‐L1 monoclonal antibody enhances the antitumour efficacy against castration‐resistant prostate cancer
Source: Clin Transl Med. 2022 Jun 13;12(6):e901. doi: 10.1002/ctm2.901 (PMC9191826; doi:10.1002/ctm2.901)
Supplement: Supplementary file 6 — Supporting Information [file CTM2-12-e901-s005.docx]

**Supplementary material**

**The construction of anti-PSMA CAR engineered NK-92 cells show effective cytotoxicity against CRPC in a mouse model even after irradiation**

**Materials and Methods**

**Cell lines and culture**

C4-2 cells (human CRPC cell line expressing PSMA), PC-3 (PSMA-negative human PCa cell line), SKOV3 (PSMA-negative ovarian cancer cell lines), NK-92 cells (human malignant non-Hodgkin’s lymphoma natural killer cell line), and 293T cells (human embryonic kidney cell line) were obtained from the American Type Culture Collection (ATCC). C4-2 cells expressing firefly luciferase (C4-2-luc) were obtained by viral transduction of lentivirus carrying pLV-luciferase neoplasmid kindly provided by Ping Guo (Allife Medicine INC. Beijing, China). The culture conditions of PCa and ovarian cancer cell lines, NK-92, CAR NK-92 and 293T cells were mentioned in our main manuscript.

**Generation of anti-PSMA-CAR NK-92 cells**

We developed a CAR construction targeting human PSMA based on a novel and high affinity specific polypeptide selected using Ph.D.-12 Phage Display Peptide Library Kit (NEB). Ph.D. peptide libraries (12-mer) were purchased from New England Biolabs 165 (Ipswich, MA, USA). The phage libraries were amplified and titrated according to the manufacturer’s instructions. A total of 3 plaques with the highest affinity to the PSMA recombinant protein were selected for sequencing using Ph.D.™-12 Phage Display Peptide Library, the amino acid sequences of the 3 selected peptides used for the CAR construction were listed as follows: KHLHYHSSVRYG (KHL); WTNHHQHSKVRE (WTN); GTIQPYPFSWGY(GTI). Each peptide segment was crossed by two repeats, and the interpeptide segments were separated by a linker: *GGT ACC ATC CAA CCA TAT CCA TTT TCC TGG GGG TAC (GTI) GGT GGT GGT GGT AGT (linker) AAA CAT CTC CAT TAT CAT TCT TCG GTT AGG TAC GGT (KHL) GGC GGC GGC GGC AGC (linker) TGG ACA GAT CAT CAC CAA CAT TCC AAA GTT CGT GAG (WTN) GGA GGA GAT GAC TTT GGA GGA AGC (linker) GGA ACA ATA CAG CCT TAC CCT TTC TCG TGG GGC TAT (GTI) GGG GGG GGG GGG AGT (linker) AAG CAC CTG CAC TAC CAC TCC TCA GTC AGA TAT GGA (KHL) GGA GGC GGT GGG AGC (linker) TGG ACT GAC CAC CAT CAG CAC TCG AAG GTA CGA GAA (WTN).*

The intracellular signaling domains of CAR were based on the full human 2B4 (CD244) sequence, and the retroviral vector pLenti-CMV/TO-eGFP-Puro (w159-1 addgene) was chosen as the skeleton frame. The extracellular segment of the CAR constructed by the 3 selected peptides or anti-mesothelin antibody scFv (SS1)-PE38 [1] substituted the segment of eGFP. The full-length human NKG2D co-stimulatory domain was fused to the CD244 signaling domain of natural killer cells (**Fig. S1a**). Then we used the CAR lentiviral transfer vector to produce viral particles in 293 T cells. A mixture of pLenti-CMV/TO-CAR-Puro, PMD2.0G, and pspaX2 plasmid was added to 293T cells and incubated in DMEM medium. To acquire the viral particles, the supernatant was collected every 24 h with freshly added DEME, centrifuged at a speed of 5×10^4^ g for 2 h. Then, NK-92 cells were infected with lentivirus carrying pLenti-CMV/TO-CAR-Puro or empty vector (VEC), viral particles were suspended in DPBS to transfect 3×10^5^ NK-92 cells in 2ml MEMα in the presence of polybrene (4 μg/mL, Sigma-Aldrich). 2 μg/ml of puromycin was added to the cell culture medium to filtrate uninfected cells. After CAR transduction by viral particles, a subset of stable CAR-positive NK-92 cell was picked up and cloned. The established anti-PSMA-CAR NK-92 included a PSMA-targeted peptide, a transmembrane domain (CD244) and intracellular domain (CD244 and NKG2D co-stimulatory domain) (**Fig. S1b**). To validate the CAR expression, we stained CAR NK-92 cells with PSMA, followed by a flow cytometry analysis.

**Irradiation of** **CAR NK-92 cells**

CAR NK-92 cells and parental NK-92 cells were collected and resuspended in fresh culture medium before irradiation. Cells mentioned in T75 culture flask were irradiated with 5 Gy or 10 Gy using an X-ray irradiator (Varian unique, USA) at room temperature. The distance between the radiation source and the cells was 100 cm, the height of the liquid level was 0.5cm, and the radiation area was set as 30cm×30cm (**Fig. S2**). The proliferation of CAR NK-92 and NK-92 cells was evaluated by counting viable cells using trypan blue exclusion daily for one week. Cells were cultured for 48h and used for subsequent *in vitro* cytotoxicity assays and *in vivo* experiments.

**CCK-8 assay**

Cytotoxicity *in vitro* of irradiated and unirradiated CAR NK-92 cells was assessed by Cell Counting Kit-8 (CCK-8, Dojindo, Japan). C4-2/PC3/SKOV3 cells in logarithmic growth phase were added to a 96-well plate with 10, 000 cells/ well respectively. On the second day, effector cells were added as an effector to the target ratio (E/T) of 1:1 and tested in triplicate. The CCK-8 solution was added to the cells at 6 h, 12 h and 24 h, and the mixture was then incubated for 4 h at 37 °C. The inhibition ratio was used to evaluate cytotoxicity of effectors as described in our main manuscript. The target cells included C4-2 cells (PSMA (+) Mesothelin (-)), PC3 cells (PSMA (-) Mesothelin (-)), and SKOV3 cells (PSMA (-) Mesothelin (+)). CAR NK-92 and VEC NK-92 cells were irradiated with 5 Gy or 10 Gy before studying the function of NK-92 cells. Unirradiated NK-92 cells were used as controls

**Cytokine release assay**

Briefly, a different number of effector cells were seeded in triplicate together with 1x10^4^ C4-2 cells, in 96-well plates. Cytokine secretion was measured after 12 h of incubation using the Human ELISA INF-γ kits (BioLegend). Negative and positive controls were represented by NK-92 cells that remained unstimulated or were treated with 10 μg/ml of PHA-M (Sigma-Aldrich), respectively. The concentrations of interleukin (IL)-6 in serum collected from mice tail vein were determined using a human IL-6 ELISA Kit (invitrogen).

**Flow cytometry**

As described in main manuscript, the 5 Gy-irradiated CAR NK-92 cells were collected after cocultured with C4-2 cells (E/T=1:1), and immunostained with CD25 PE and CD69 PE purchased from BioLegend for further analysis.

**Mice and procedures**

NOD/SCID mice aged 5 weeks were purchased from Beijing Vital River Laboratory Animal Technology Company. For *in vivo* experiments testing anti-PSMA-CAR NK-92 cell function against CRPC, 1×10^7^ C4-2-luc cells were suspended in PBS (1 × 10^7^/ml) and then injected into the peritoneal cavity using 1 ml Omnican syringe with 30G gaμge needle (B.Braun Melsungen AG,OPM, Germany). Bioluminescent intensity (BLI) was measured 4 days after injection of C4-2-luc cells to evaluate tumor induction and growth. Mice were imaged 15min following intraperitoneal injection (i.p.) of luciferin (150mg/ml, Dalian Meilun Biotech Co., Ltd, China), and the BLI was measured using IVIS Lumina Series III (PerkinElmer, USA). The body weight of mice was measured every 3 or 4 days. Based on BLI and body weight, mice were randomly divided into four groups (n=6): Control, NK-92, VEC NK-92, and anti-PSMA-CAR NK-92 group. 2×10^7^ NK-92 cells suspended in 500ul of PBS were injected via ip, while control group received PBS of the same volume ip. In addition to tumor progression evaluated by BLI, survival was monitored in mice.

**Results**

**Irradiated** **anti-PSMA-CAR NK-92 cells have no proliferation ability after a suitable dose of radiation and retain a high and specific cytotoxic ability similar to their unirradiated counterparts *in vitro***

The construction of the established anti-PSMA-CAR NK-92 was shown in **Fig. S1b**. The flow cytometry result confirmed the expression of CAR on anti-PSMA-CAR NK-92 (**Fig. S3a**). To lay a foundation for the clinical use of CAR NK-92 cells, we tested the effects of irradiation on the proliferation and cytotoxicity of anti-PSMA-CAR NK-92 cells. The viability results demonstrated that anti-PSMA-CAR NK-92 cells did not proliferate immediately after irradiation and the viable cells were no longer detectable after 6–7 days when the radiation dose was 5 Gy and 10 Gy (**Fig. S3b**). The CCK results revealed that both the 5 Gy-irradiated and unirradiated anti-PSMA-CAR NK-92 cells exhibited significantly higher and more specific cytotoxicity against PSMA positive C4-2 cancer cells rather than PSMA negative cancer cells including PC3 and SKOV3, when compared with parental NK-92 cells, VEC NK-92 cells, and anti-Mesothelin-CAR NK-92 cells (P<0.05) (**Fig. S3c, d, and e**), and the cytotoxic effect of 5 Gy-irradiated CAR NK-92 cells on PSMA positive target cells was almost the same as that of unirradiated CAR NK-92 cells (P＞0.05). By comparison, the specific cytotoxicity of 10 Gy-irradiated anti-PSMA-CAR NK-92 cells was significantly diminished (P< 0.05). Similarly, both 5 Gy-irradiated and unirradiated anti-PSMA-CAR NK-92 cells secreted more IFN-γ than VEC NK-92 cells, when cocultured with C4-2 cells, and the secretion of IFN-γ increased significantly with prolongation of coculture time. However, IFN-γ secretion of anti-PSMA-CAR NK-92 cells when cocultured with C4-2 cells was significantly reduced after exposure to 10 Gy irradiation (P<0.05) (**Fig. S3f**). In addition, we observed that the expressions of the two activation antigens, CD69 and CD25, were significantly increased on 5 Gy-irradiated CAR NK-92 cells when cocultured with C4-2 cells (**Fig. S4**). Collectively, 5 Gy radiation inhibits proliferation of CAR NK-92 cells, and does not alter the cytotoxicity against target cells or cytokine IFN-γ secretion, thus demonstrating that 5 Gy is a suitable radiation dose to ensure the safety and effectiveness of anti-PSMA-CAR NK-92 cells.

**The 5 Gy-irradiated anti-PSMA-CAR NK-92 cells significantly controlled the growth of the CRPC tumor and prolonged the survival time of mice *in vivo***

The exact treatment schedule was shown in **Fig. S5a**. Sixty NOD/SCID mice were inoculated with 1×10^7^ C4-2-luc cells via intraperitoneal injection, 4 days later, the mice were randomly divided into 4 groups according to their BLI (**Fig. S5b**): Control (#11,19,23,45,57,58), NK-92 (#3,6,7,8,12,14), VEC NK-92 (#2,9,10,15,16,20), anti-PSMA-CAR NK-92 (#1,4,5,17,21,25). There was no significant difference in BLI between the four groups (P＞0.05) (**Fig. S5c**). At day 0, day 7, day 14 and day 21, PBS and 1×10^7^ cells of irradiated NK-92, VEC NK-92, anti-PSMA-CAR NK-92 were administered i.p. (**Fig. S5a**). On day 3, 10, 17, 21, 24, 28, 31, 38 after treatment, the growth of C4-2-luc cells in mice was dynamically monitored by BLI. The BLI of the control, NK-92, and VEC NK-92 groups was almost the same (P＞0.05), while the BLI of the anti-PSMA-CAR NK-92 group was significantly lower than that of the other three groups (P<0.05) (**Fig. S5d, e**). The results indicated that PCa progression in the anti-PSMA-CAR NK-92 group was slower than that of the other groups and the 5 Gy-irradiated anti-PSMA-CAR NK-92 cells significantly delayed CRPC tumor progression *in vivo.* Seventeen days after treatment, the body weight of the mice in four groups started to drop evidently, and there were no significant differences between the four groups before day 31. Furthermore, the body weight of the surviving mice in the anti-PSMA-CAR NK-92 group began to increase after day 35 (**Fig. S5f**). The median survival times of the control, NK-92, VEC NK-92, anti-PSMA-CAR NK-92 groups were 36, 36, 40, and 47 days, respectively. Mice in the anti-PSMA-CAR NK-92 group showed a significantly longer survival time than those of the control, NK-92, and VEC NK-92 groups (P<0.05) (**Fig. S5g**). Besides, serum IL-6 of mice in the anti-PSMA-CAR NK-92 cell treatment group was marginally higher than that of the control group (56.1± 6.2 vs.50.2± 12.0, P＞0.05) (**Fig. S5h**)

**References**

[1] Chowdhury PS, Viner JL, Beers R, Pastan I. Isolation of a high-affinity stable single-chain Fv specific for mesothelin from DNA-immunized mice by phage display and construction of a recombinant immunotoxin with anti-tumor activity. Proc Natl Acad Sci U S A. 1998; 95(2):669-74.

**Legends**

**Figure S1:** Structure diagram of anti-PSMA-CAR plasmid and anti-PSMA-CAR NK 92. **a.** Structure diagram of the lentiviral vector anti-PSMA peptide-CD244-NKG2D. (GTI, coding sequence of Glycine-Threonine-Isoleucine; KHL: coding sequence of Lysine-Histidine-Leucine; WTN: coding sequence of Tryptophan-Threonine-Asparagine; transmembrane region, transmembrane domain of CD244; CD244, cytoplasmic domain of CD244; NKG2D, cytoplasmic domain of NKG2D; Puro, puromycin resistant gene). **b.** Schematic diagram of anti-PSMA-CAR NK 92.

**Figure S2:** The radiation scene of CAR NK-92 and parental NK-92 cells. These cells were cultured in T75 culture flasks and made stand for 5 minutes before getting irradiated with 5Gy or 10Gy using an X-ray irradiator at room temperature. The distance between the radiation source and the cells was set as 100cm, the height of the liquid level was 0.5cm, and the radiation area was set as 30cm×30cm to include four T75 culture flasks each time.

**Figure S3:** The anti-PSMA-CAR NK-92 cells lose proliferation ability after exposure to 5Gy irradiation and retain a high and specific cytotoxicity against PSMA-expressing C4-2 cells *in vitro.* **a.** anti-PSMA-peptide expression on surface of anti-PSMA-CAR NK-92 cells determined by flow cytometry with labelled PSMA antigen. **b.** Viability of the anti-PSMA-CAR NK-92 cells unirradiated and 5, 10 Gy-irradiated. Viable cells were counted using trypan blue exclusion at the indicated time points. **c.** The cytotoxicity of 0, 5 and 10 Gy-irradiated anti-PSMA-CAR NK-92 cells and anti-mesothelin-CAR NK-92 cells against PSMA (-) Mesothelin (-) PC3 cells assessed by CCK-8 assays at the indicated time points. **d.** The cytotoxicity of 0, 5 and 10 Gy-irradiated anti-PSMA-CAR NK-92 cells and anti-mesothelin-CAR NK-92 cells against PSMA (-) Mesothelin (+) SKOV3 cells assessed by CCK-8 assays at the indicated time points. **e.** The cytotoxicity of 0, 5 and 10 Gy-irradiated anti-PSMA-CAR NK-92 cells and anti-mesothelin-CAR NK-92 cells against PSMA (+) Mesothelin (-) C4-2 cells assessed by CCK-8 assays at the indicated time points. **f.** IFN-γ secretion by 0, 5 and 10 Gy-irradiated anti-PSMA-CAR NK-92 cells upon engagement with C4-2 prostate cancer cells at the indicated time points. The NK-92 cells alone was set as negative control while NK-92 cells stimulated with PHA-M was set as positive control. In **b-f**, unirradiated NK-92 cells and irradiated VEC CAR NK-92 cells were included for comparison. Data were shown as mean means±SD of 3 independent experiments. The ANOVA test followed by a Tukey post hoc test was used for multiple group comparisons (**b-f**). *, P<0.05; ns, not significant; PHA-M, phytohemagglutinin-M type.

**Figure S4:** The expression of activation markers CD25 and CD69 on surface of CAR NK-92 cells cocultured with C4-2 cells at the indicated time points. **A**, Representative flow cytometry plots (n=3) showing expression of CD25 on CAR NK-92 cells incubated with C4-2 cells for 0, 6, 12, 24 h. **B**, Representative flow cytometry plots (n=3) showing expression of CD69 on CAR NK-92 cells incubated with C4-2 cells for 0, 6, 12, 24 h. **C**, Summary data (n=3) of MFI for CD25 and CD69 of CAR NK-92 cells at the indicated time points. The ANOVA test followed by a Tukey post hoc test was used for multiple group comparisons (**C**) *, P<0.05; ns, not significant.

**Figure S5:** Antitumor activity of anti-PSMA-CAR NK 92 cells against prostate cancer in a C4-2-luc cells peritoneal disseminated mouse model**. a.** Experimental schedule. C4-2-luc cells were injected into peritoneal cavity of NOD/SCID mice. Four days later, mice received ip injections of NK-92 cells, VEC NK-92 cells, or anti-PSMA-CAR NK 92 cells once a week for 4 weeks. Control mice were treated with PBS and tumor progression was monitored by BLI. **b.** Bioluminescence images of all mice collected four days after transplantation. Mice were randomly divided into four treatment groups according to the average radiance of bioluminescence imaging. **c.** Statistical analysis of baseline BLI data in the control, NK-92, VEC NK-92, and anti-PSMA-CAR NK-92 groups before treatment (n=6, ANOVA). **d.** Bioluminescence images of all four groups collected on indicated days after treatment. **e.** Statistical analysis of BLI data in groups (n = 6, ANOVA). **f.** Body weight of all groups on indicated days (n = 6, ANOVA). **g.** Cumulative Kaplan-Meier survival curves for mice (n = 6, log-rank test). **h.** ELISA data showing serum IL-6 of mice in the anti-PSMA-CAR NK-92 cells treatment group and the control group (n=6, unpaired t test). Data were expressed as the means±SD, *, P <0.05; NS, not significant; BLI, bioluminescence intensity; ANOVA, analysis of variance; ELISA, enzyme-linked immunosorbent assay.
